# The Role of Lifestyle Behaviors in the Association Between Behavioral Procrastination and Metabolic Syndrome: A Cross-Sectional Study of 92,184 Workers

**DOI:** 10.3390/nu18213678

**Published:** 2026-11-06

**Authors:** Manuel Sarmiento Cruz, Pedro Juan Tárraga López, Mónica Silu Piña Dabreu, Lluis Rodas Cañellas, Ángel Arturo López-González, José Ignacio Ramírez-Manent

**Affiliations:** 1 Balearic Islands Health Service, 07010 Palma, Spain; msarmiento1979@gmail.com (M.S.C.);; 2 Faculty of Medicine, University of Castilla La Mancha (UCLM), 02008 Albacete, Spain; pjtarraga@sescam.jccm.es; 3 ADEMA-University School, University of the Balearic Islands, 07009 Palma, Spain

**Keywords:** procrastination, metabolic syndrome, life style, exercise, diet, Mediterranean, smoking

## Abstract

**Background:** Procrastination has been associated with unfavorable health behaviors and poorer health outcomes, but the extent to which lifestyle-related characteristics statistically overlap with the association between procrastination and metabolic syndrome (MetS) remains unclear. This study examined the cross-sectional association between operational Pure Procrastination Scale-9 (PPS-9) categories and MetS and quantified changes in this association after incorporation of physical activity, Mediterranean diet adherence, and smoking. **Methods:** A cross-sectional study was conducted among 92,184 Spanish workers aged 18–69 years undergoing occupational health examinations between 2021 and 2024. Participants were classified into four operational, non-clinical PPS-9 categories: very low/absent, moderate, high, and very high. MetS was primarily defined according to Joint Interim Statement criteria. Sequential logistic regression models were adjusted for age, sex, education, social class, physical activity, Mediterranean diet adherence, and current smoking. Statistical attenuation was calculated on the log-odds scale relative to the sociodemographic-adjusted model. Interaction and sensitivity analyses were also performed. **Results:** MetS prevalence increased progressively across operational PPS-9 categories, from 0.4% in the very low/absent category to 5.2%, 16.7%, and 37.3% in the moderate, high, and very high categories, respectively. Higher PPS-9 categories were also associated with markedly lower physical activity and Mediterranean diet adherence. After adjustment for age, sex, education, and social class, higher operational PPS-9 categories remained associated with greater odds of prevalent MetS. However, the magnitude of the odds ratios was influenced by the exceptionally low MetS prevalence in the reference category, and these estimates should not be interpreted as prevalence ratios. Marginally standardized analyses showed that adjustment for physical activity and Mediterranean diet adherence reduced absolute risk differences between PPS-9 categories, with larger reductions following adjustment for physical activity. In the fully adjusted model, absolute risk differences relative to the very low/absent category were 3.76, 10.95, and 22.31 percentage points for the moderate, high, and very high categories, respectively. These findings were consistent with the descriptive pattern of the sequential logistic models, while avoiding reliance solely on changes in logistic regression coefficients. Significant statistical interactions were observed for physical activity and Mediterranean diet adherence. The graded association remained consistent across multiple sensitivity analyses. **Conclusions:** Higher operational PPS-9 categories were strongly associated with less favorable lifestyle profiles and a higher prevalence of MetS in this large occupational population. Physical activity and Mediterranean diet adherence showed substantial statistical overlap with the PPS-9–MetS association, although a graded association remained after multivariable adjustment. These cross-sectional findings do not establish temporal, mediational, or causal relationships and warrant confirmation in longitudinal studies.

## 1. Introduction

Procrastination is generally defined as the voluntary delay of an intended course of action despite the expectation that the delay may have negative consequences [1]. It is commonly conceptualized as a form of self-regulatory failure and has traditionally been investigated in relation to academic performance, occupational functioning, and psychological well-being [1,2]. However, increasing attention has been directed toward its potential relevance to physical health and health-related behaviors. From this perspective, procrastination may extend beyond the postponement of academic or work-related tasks and coexist with difficulties in initiating or maintaining behaviors relevant to long-term health [3,4].

A growing body of research has reported associations between procrastination and less favorable health and lifestyle profiles. Higher procrastination has been associated with greater psychological distress, poorer self-rated health, sleep problems, and less favorable health behaviors [2,3,4,5]. The procrastination–health model has proposed stress and health behaviors as two potentially relevant components of the relationship between procrastination and health [3,4]. More recent longitudinal evidence has contributed to this literature. In a cohort of Swedish university students, higher procrastination was associated with several subsequent health outcomes, including greater symptoms of depression, anxiety, and stress, as well as poor sleep quality and physical inactivity [5]. Not all health behaviors showed consistent associations, however, emphasizing that the relationship between procrastination and lifestyle is likely to be heterogeneous across behaviors and populations. These findings support further investigation of the behavioral characteristics accompanying procrastination while highlighting the importance of distinguishing statistical associations from causal relationships.

Lifestyle-related factors are particularly relevant in the context of cardiometabolic health. Physical inactivity, smoking, and unfavorable dietary patterns frequently coexist with metabolic abnormalities and are associated with an adverse cardiovascular risk profile [6,7]. Metabolic syndrome (MetS), characterized by the clustering of central adiposity, elevated blood pressure, impaired glucose regulation, hypertriglyceridemia, and reduced high-density lipoprotein cholesterol, provides an integrative framework for examining cardiometabolic health [6,7]. Because health-related behaviors require repeated initiation, planning, and maintenance over time, differences in self-regulatory patterns may coexist with differences in these behaviors. Nevertheless, particularly in cross-sectional settings, the simultaneous presence of procrastination, unfavorable lifestyle characteristics, and metabolic abnormalities cannot establish either temporal ordering or a causal pathway among these factors.

Recent findings from our large occupational population have extended the study of behavioral procrastination into the cardiometabolic domain. Higher operational Pure Procrastination Scale-9 (PPS-9) categories were associated with a progressively less favorable cardiometabolic profile and a higher prevalence of MetS [8]. Subsequent analyses in the same occupational population showed that higher operational PPS-9 categories were also associated with multiple surrogate markers of insulin resistance and visceral adiposity [9]. Importantly, adjustment for demographic and lifestyle-related characteristics substantially attenuated several of these associations, suggesting considerable overlap between procrastination categories, participant characteristics, health behaviors, and metabolic profiles [9]. In addition, higher procrastination was associated with greater heart age acceleration and a less favorable cardiovascular aging profile in this occupational population [10]. Taken together, these findings indicate that operational PPS-9 categories identify groups with markedly different cardiometabolic characteristics, while raising the question of the extent to which differences in lifestyle-related factors statistically account for part of these cross-sectional associations.

The present study uses exactly the same analytical sample of 92,184 workers and the same underlying occupational health examination data as the three previous investigations [8,9,10]; therefore, participant overlap is complete (100%), and this is a secondary analysis of an existing dataset rather than an independent cohort or replication sample. The earlier MetS study [8] established the overall association between operational PPS-9 categories and MetS, whereas the subsequent studies examined insulin resistance and visceral adiposity phenotypes [9] and heart age acceleration [10]. The present manuscript does not claim these previously reported associations as new findings. Instead, its distinct contribution is the separate evaluation of physical activity, Mediterranean diet adherence, and smoking in sequentially adjusted models of the procrastination–MetS association; the assessment of statistical interactions with lifestyle characteristics; and the estimation of marginally standardized MetS prevalences and absolute prevalence differences. These analyses address a different research question using the same participants and previously collected variables.

Despite the growing evidence linking procrastination with both health behaviors and cardiometabolic characteristics, the role of lifestyle-related factors in the association between behavioral procrastination and MetS remains insufficiently characterized. In particular, it is unclear to what extent differences in physical activity, dietary habits, and smoking across operational PPS-9 categories account statistically for the observed association with MetS after major demographic and socioeconomic characteristics are considered. Examining changes in the magnitude of this association after the sequential incorporation of lifestyle-related variables may help determine the degree to which the procrastination–MetS relationship overlaps with differences in behavioral profiles. In a cross-sectional study, such attenuation can be quantified and compared across behavioral factors without assuming that these variables constitute causal mediators.

Therefore, the primary aim of the present study was to examine the extent to which physical activity, dietary habits, and smoking statistically account for the cross-sectional association between operational PPS-9 categories and MetS in a large population of Spanish workers. A secondary aim was to assess the relative contribution of individual lifestyle-related factors to the attenuation of this association and to explore whether the magnitude of the association between PPS-9 category and MetS differed according to selected lifestyle characteristics. Given the cross-sectional design, all analyses were intended to characterize statistical associations, overlap between behavioral and cardiometabolic profiles, and patterns of attenuation rather than to establish temporal relationships, causal pathways, or mediation effects.

## 2. Materials and Methods

### 2.1. Study Design and Population

A multicenter cross-sectional observational study was conducted using data obtained from routine occupational health examinations performed between January 2021 and December 2024 in different Spanish regions. The study population consisted of active workers aged 18–69 years undergoing standardized occupational health assessments as part of preventive workplace health surveillance programs. The analytical dataset did not retain identifiers for the participating regions, occupational health centers, or workplaces, preventing a more detailed description of their geographical and institutional distribution. Workers were included from the available occupational health examination records, subject to the eligibility and completeness criteria described below; therefore, the sample should not be considered a probability sample of the Spanish working population. The study population included workers from a broad range of economic activities, covering nearly all occupational sectors. Occupational information was recorded in the analytical dataset, although a reliable sector-by-sector breakdown could not be reconstructed from the available coding information. Examination dates and individual longitudinal identifiers were not retained. Consequently, it was not possible to independently verify whether repeated examinations from the same worker were present or to establish a retrospective observation-selection rule. Similarly, clustering within centers or workplaces and variation by examination year could not be evaluated.

**Inclusion criteria.** Participants were eligible for inclusion if they were active workers aged 18–69 years who underwent an occupational health examination between January 2021 and December 2024 and had an examination record available in the occupational health database used for this secondary analysis.

**Exclusion criteria.** Records were excluded from the analytical sample if information was missing for behavioral procrastination, anthropometric measurements, fasting biochemical parameters, or the lifestyle and sociodemographic variables required for the analyses.

The initial database included 94,327 workers. Participants with missing information on behavioral procrastination, anthropometric measurements, fasting biochemical parameters, or key lifestyle and sociodemographic variables required for the present analyses were excluded. A total of 2143 records (2.3%) had incomplete information in one or more of these variables and were therefore excluded, resulting in a final analytical sample of 92,184 workers (97.7%). The participant selection process is summarized in Supplementary Figure S1.

No a priori sample size calculation was performed because the present study was a secondary analysis of an existing occupational health database rather than a prospectively recruited study. The sample size was determined by the number of available records meeting the predefined eligibility and complete-case criteria during the study period. No additional participants were recruited specifically for this analysis.

Individual-level data for the 2143 excluded records were not retained in the analytical dataset; therefore, their demographic, occupational, lifestyle, and metabolic characteristics could not be described or compared with those of the included participants.

The study was conducted and reported in accordance with the Strengthening the Reporting of Observational Studies in Epidemiology (STROBE) recommendations for observational research [11]. Given the cross-sectional design, all analyses were intended to characterize associations among behavioral procrastination, lifestyle-related factors, and metabolic syndrome (MetS) at the time of assessment. No temporal sequence or causal relationship among these variables was assumed.

The present study represents a secondary analysis of the same occupational health dataset used in three previous investigations [8,9,10]. The analytical sample comprises exactly the same 92,184 participants, corresponding to 100% participant overlap. No additional participants or independent examinations were recruited specifically for the present analysis. The novelty of this study lies in the analytical objectives and statistical approaches rather than in the study population or the underlying data.

### 2.2. Ethical Considerations

All examinations were performed by trained occupational health professionals following standardized protocols. The study was conducted in accordance with the ethical principles of the Declaration of Helsinki [12] and was approved by the Research Ethics Committee of the Balearic Islands (CEI-IB 4383/20 PI; approved on 26 November 2020). As the study used anonymized secondary data obtained during routine occupational health examinations, the requirement for informed consent was waived. Personal identifiers were removed before statistical analysis to ensure participant confidentiality, and data processing complied with applicable European and Spanish regulations on personal data protection.

### 2.3. Assessment of Behavioral Procrastination

Behavioral procrastination was assessed using a nine-item Spanish-language questionnaire adapted from the conceptual content of the Pure Procrastination Scale (PPS), originally developed by Steel [13]. The operational score classification is presented in Supplementary Table S1, and the conceptual correspondence between the administered items and the original instrument is presented in Supplementary Table S2. The questionnaire was translated into Spanish for use during routine occupational health examinations, with the participation of a psychologist and another professional in the translation process. It was self-administered by participants during these examinations. The complete Spanish-language questionnaire, including the exact wording of the nine items, participant instructions, response options, and scoring procedure, is provided in Supplementary Material Section S1.1.

The questionnaire comprises nine statements assessing procrastination-related behaviors, including postponement of decisions (items 1, 4, and 9), delay in task initiation (items 2, 5, and 8), and difficulties meeting deadlines (items 3, 6, and 7). These three domains represent conceptual groupings of the items rather than empirically validated subscales in the present population. Previous research has examined the measurement properties of procrastination scales across European populations [14] Supplementary Material Section S1.2.

Each item was rated on a five-point Likert scale ranging from 1 (“completely disagree”) to 5 (“completely agree”). The total PPS-9 score was calculated as the sum of the nine items and ranged from 9 to 45 points, with higher scores indicating greater behavioral procrastination.

For the present analyses, participants were classified into four operational categories according to their total PPS-9 score: very low/absent (9–17 points), moderate (18–26 points), high (27–35 points), and very high (36–45 points). These score ranges were recommended by the psychologist involved in the questionnaire adaptation and were established before the metabolic outcomes were examined. They divide the possible total score range (9–45 points) into four approximately equal-width intervals to provide a descriptive classification of increasing procrastination-related behaviors. The cut-points were not derived from empirical optimization, normative reference values, or validated clinical thresholds. Accordingly, these categories are operational groupings specific to the present study and should not be interpreted as clinically validated, diagnostic, or prognostic cut-off points Supplementary Material Section S1.3 and Table S1.

Although the translation process involved professional participation, the specific nine-item Spanish-language version used in this occupational population has not undergone formal psychometric validation. Therefore, its internal consistency, factorial validity, and measurement equivalence with the original instrument have not been established in this population.

Furthermore, the available analytical dataset retained only the final operational PPS-9 category, without individual item responses or continuous total scores. Consequently, internal consistency coefficients, such as Cronbach’s alpha or McDonald’s omega, and exploratory or confirmatory factor analyses could not be calculated retrospectively. Analyses using continuous PPS-9 scores were likewise not possible.

Accordingly, the four groups are referred to throughout the present study as operational PPS-9 categories. Operational PPS-9 category constituted the main exposure variable in the statistical analyses, with the very low/absent category serving as the reference group.

### 2.4. Anthropometric and Clinical Assessment

Anthropometric and clinical measurements were obtained by trained occupational health professionals following standardized procedures.

Body weight was measured using calibrated electronic scales, with participants wearing light clothing and no shoes. Height was measured using a stadiometer under standardized conditions. Body mass index (BMI) was calculated as body weight in kilograms divided by height in meters squared (kg/m^2^).

Waist circumference was measured using a non-elastic measuring tape at the midpoint between the lower costal margin and the iliac crest, with participants standing and after normal expiration.

Blood pressure was measured in the seated position after a minimum resting period of five minutes using validated automatic sphygmomanometers. When two measurements were available, the mean value was used for analysis.

### 2.5. Biochemical Measurements

Venous blood samples were collected after overnight fasting. Biochemical determinations included fasting plasma glucose, triglycerides, total cholesterol, high-density lipoprotein cholesterol (HDL-C), and low-density lipoprotein cholesterol (LDL-C). Laboratory determinations were performed in certified clinical laboratories using standardized automated methods and internal quality-control procedures.

Glucose and lipid concentrations were expressed in mg/dL. Anthropometric, clinical, and biochemical measurements were used to determine the presence of MetS according to the definitions described below.

### 2.6. Definition of Metabolic Syndrome

Metabolic syndrome was the primary cardiometabolic outcome of the study. For the primary analysis, MetS was defined according to the harmonized Joint Interim Statement (JIS) criteria [15]. Participants were classified as having MetS when at least three of the following five components were present:

- Waist circumference ≥ 94 cm in men or ≥80 cm in women;
- Triglycerides ≥ 150 mg/dL;
- HDL-C < 40 mg/dL in men or <50 mg/dL in women;
- Systolic blood pressure ≥ 130 mmHg or diastolic blood pressure ≥ 85 mmHg;
- Fasting plasma glucose ≥ 100 mg/dL.

The JIS definition was selected as the primary outcome because it provides a harmonized definition based on the presence of any three of the five metabolic abnormalities without requiring central obesity as a mandatory component [15].

To assess whether the observed associations were dependent on the definition of MetS, alternative classifications according to the revised Adult Treatment Panel III (ATP III) and International Diabetes Federation (IDF) criteria were examined in sensitivity analyses [16,17]. The ATP III definition required at least three of the five metabolic components, using waist circumference thresholds of >102 cm in men and >88 cm in women. The IDF definition required central obesity, defined using European waist circumference thresholds of ≥94 cm in men and ≥80 cm in women, plus at least two of the remaining four components. For all three definitions, elevated blood pressure was identified when either systolic blood pressure was ≥130 mmHg or diastolic blood pressure was ≥85 mmHg. The analytical dataset did not contain treatment-specific medication variables; therefore, MetS classification was based on measured metabolic components, without incorporating medication-based criteria, including treatment for reduced HDL-C. The IDF classification was recalculated using the European waist circumference thresholds.

### 2.7. Lifestyle-Related Variables

Smoking status, physical activity, and adherence to the Mediterranean diet were assessed using standardized questionnaires administered as part of the occupational health assessment. These variables were examined as lifestyle-related factors associated with operational PPS-9 categories and MetS. Given the cross-sectional design, they were treated as lifestyle-related covariates and were not assumed to represent causal mediators.

#### 2.7.1. Smoking Status

Smoking status was assessed by self-report using the standardized occupational health questionnaire. For the present analyses, participants were classified into two mutually exclusive categories: current smokers and non-smokers. The non-smoker category included both never smokers and former smokers. This binary classification was used to characterize current smoking status while retaining participants with previous smoking exposure within the non-current smoking category [18,19].

Information on daily cigarette consumption was additionally available for current smokers and was categorized as <10 cigarettes/day, 10–20 cigarettes/day, or >20 cigarettes/day. Smoking status (current smoker vs. non-smoker) rather than cigarette consumption was used as the principal smoking-related variable in the multivariable models.

#### 2.7.2. Physical Activity

Physical activity was assessed using the short form of the International Physical Activity Questionnaire (IPAQ-SF), a validated instrument for the assessment of habitual physical activity in epidemiological studies [20].

The IPAQ-SF collects information on the frequency and duration of vigorous-intensity activity, moderate-intensity activity, and walking performed during the previous seven days. Responses were processed according to standard IPAQ scoring and data-cleaning procedures, and total physical activity was expressed as metabolic equivalent minutes per week (MET-min/week).

For the present analyses, participants accumulating ≥600 MET-min/week were classified as physically active, whereas those accumulating <600 MET-min/week were classified as physically inactive [20,21]. This dichotomous classification was used in the primary analyses.

#### 2.7.3. Mediterranean Diet Adherence

Adherence to the Mediterranean diet was assessed using the 14-item Mediterranean Diet Adherence Screener (MEDAS), originally developed within the PREDIMED study and subsequently validated for the rapid assessment of adherence to a Mediterranean dietary pattern [22,23,24].

The questionnaire assesses habitual dietary practices and consumption of key components of the Mediterranean dietary pattern, including the use of olive oil and the consumption of vegetables, fruits, legumes, nuts, fish, meat products, and other relevant food groups. Each item contributes 0 or 1 point, yielding a total MEDAS score ranging from 0 to 14 points, with higher scores indicating greater adherence to the Mediterranean dietary pattern.

For the present analyses, participants with a MEDAS score ≥9 were classified as having high adherence to the Mediterranean diet, whereas those scoring <9 were classified as having low adherence [22,23,24]. This dichotomous classification was used in the primary analyses.

Smoking status, physical activity, and Mediterranean diet adherence were examined individually and jointly in the multivariable models. Changes in the association between operational PPS-9 categories and MetS after incorporation of these variables were interpreted as statistical attenuation of the observed cross-sectional association and not as evidence of mediation, indirect effects, temporal pathways, or causal relationships.

### 2.8. Sociodemographic and Occupational Variables

Age, sex, educational level, and social class were considered sociodemographic covariates.

Age was analyzed as a continuous variable. Because previous analyses of this occupational population identified substantial differences in age distribution across operational PPS-9 categories, particular attention was given to age adjustment and age-restricted sensitivity analyses.

Sex was recorded as male or female. Educational level was classified into primary, secondary, or university education.

Social class was classified into three categories according to the occupational social class classification proposed by the Spanish Society of Epidemiology (CSO-SEE12), based on the Spanish National Classification of Occupations 2011 (CNO-2011). Class I included managers and professionals; class II included intermediate occupations and self-employed workers; and class III included manual workers. This three-category grouping was derived from the occupational classification described by Domingo-Salvany et al. [25]. Occupational activity according to the Spanish National Classification of Economic Activities (CNAE) was also available; however, social class was selected for the primary adjustment models as a more parsimonious indicator of socioeconomic and occupational position.

Information on diagnosed psychiatric disorders, including depression, anxiety disorders, and attention-deficit/hyperactivity disorder (ADHD), as well as psychotropic medication use, was not systematically available in the occupational health dataset and therefore could not be included as covariates.

### 2.9. Statistical Analysis

Continuous variables were summarized as mean ± standard deviation (SD) or median and interquartile range, as appropriate, whereas categorical variables were expressed as absolute frequencies and percentages.

Participant characteristics were described across the four operational PPS-9 categories. Continuous variables were compared using one-way analysis of variance (ANOVA) or the corresponding non-parametric test when appropriate, whereas categorical variables were compared using Pearson’s chi-square test.

Given the large sample size, statistical significance was interpreted together with the magnitude and precision of the estimates rather than relying exclusively on *p*-values.

All regression analyses were interpreted within the cross-sectional nature of the study. Regression coefficients, odds ratios, changes in coefficients across sequential models, and interaction terms were considered measures of statistical association and were not interpreted as estimates of causal effects.

#### 2.9.1. Association Between Operational PPS-9 Categories and Lifestyle-Related Variables

The distribution of physical activity, Mediterranean diet adherence, and current smoking was first described across the four operational PPS-9 categories.

Separate binary logistic regression models were subsequently used to evaluate the association between operational PPS-9 category and each lifestyle-related variable after adjustment for age, sex, educational level, and social class.

The dependent variables were physical activity (active vs. inactive), Mediterranean diet adherence (high vs. low adherence), and smoking status (current smoker vs. non-smoker). Adjusted odds ratios (aORs) and 95% confidence intervals (95% CIs) were reported, with the very low/absent PPS-9 category serving as the reference group.

#### 2.9.2. Association Between Operational PPS-9 Categories and Metabolic Syndrome

Binary logistic regression models were used to evaluate the cross-sectional association between operational PPS-9 category and the presence of MetS. Odds ratios (ORs) and 95% CIs were estimated using the very low/absent PPS-9 category as the reference group.

A sequential adjustment strategy was applied to examine changes in the magnitude of the association after incorporation of sociodemographic and lifestyle-related variables.

The models were specified as follows:

Model 1: age and sex.

Model 2: Model 1 + educational level + social class.

Model 3A: Model 2 + physical activity.

Model 3B: Model 2 + Mediterranean diet adherence.

Model 3C: Model 2 + smoking status.

Model 4: Model 2 + physical activity + Mediterranean diet adherence + smoking status.

This sequential modeling strategy was used to compare the magnitude of the cross-sectional association between operational PPS-9 categories and MetS before and after incorporation of each lifestyle-related variable separately and all three variables simultaneously.

Changes in estimates across models were interpreted exclusively as statistical attenuation of the observed cross-sectional association. They were not interpreted as evidence of mediation, indirect effects, mechanisms, temporal pathways, or causal relationships.

BMI was not included in the primary sequential models because adiposity is closely related to both the definition and metabolic expression of MetS, and its inclusion would change the estimand being evaluated. Additional adjustment for BMI was therefore examined separately as a sensitivity analysis.

To complement the odds ratios obtained from logistic regression, adjusted prevalence ratios (aPRs) were estimated using Poisson regression with a log link and robust sandwich variance estimation. The analyses used the same covariate specifications as the corresponding logistic regression models, allowing comparisons between the two association measures. Prevalence ratios were interpreted as measures of cross-sectional association, not as estimates of causal effects. Particular caution was applied to estimates involving the very low/absent PPS-9 reference category because of its unusually low prevalence of MetS and current smoking.

#### 2.9.3. Quantification of Statistical Attenuation

The degree of attenuation of the association after inclusion of lifestyle-related variables was quantified on the logistic regression coefficient scale rather than directly from the ORs.

For each operational PPS-9 category, percentage attenuation was calculated as:

Percentage attenuation (%) = 100 × (βBase − βAdjusted)/βBase

where βBase represents the logistic regression coefficient obtained from Model 2 and βAdjusted represents the corresponding coefficient after incorporation of the lifestyle-related variable or variables.

Attenuation was calculated separately after inclusion of physical activity, Mediterranean diet adherence, smoking status, and all three lifestyle-related variables simultaneously.

These percentages were interpreted exclusively as descriptive measures of the change in the estimated cross-sectional association following statistical adjustment. They were not considered estimates of a mediated proportion, indirect effect, causal contribution, or proportion of the association explained by lifestyle behaviors.

Because comparisons of logistic regression coefficients across nested models may be affected by non-collapsibility and differences in model scaling, percentage changes in log-odds coefficients were not considered directly comparable measures of the contribution of lifestyle-related variables. To complement these descriptive comparisons, we calculated marginally standardized predicted probabilities of MetS for each operational PPS-9 category under Models 2, 3A, 3B, 3C, and 4. For each fitted model, predicted probabilities were obtained by assigning each participant, in turn, to the reference PPS-9 category and to each higher category, while retaining their observed covariate values. Predictions were then averaged over the same analytical population (*n* = 92,184). Adjusted risk differences were calculated by subtracting the standardized probability for the reference category from that for each higher PPS-9 category and expressed in percentage points. Corresponding 95% confidence intervals were obtained using the delta method. These marginal risk differences provide comparisons on an absolute probability scale, although differences between adjusted models remain descriptive and should not be interpreted as causal contributions or mediated effects. These results are presented in Supplementary Table S3.

#### 2.9.4. Interaction Analyses

Exploratory interaction analyses were conducted to assess whether the magnitude of the cross-sectional association between operational PPS-9 category and MetS differed according to selected lifestyle characteristics.

Multiplicative interaction terms between operational PPS-9 category and physical activity, Mediterranean diet adherence, and smoking status were introduced separately into the corresponding adjusted logistic regression models.

Where evidence of statistical interaction was observed, adjusted predicted probabilities of MetS were estimated across operational PPS-9 categories according to the corresponding lifestyle characteristic to facilitate interpretation. These probabilities were calculated by marginal standardization. For each combination of PPS-9 category and lifestyle subgroup, predictions were obtained for all 92,184 participants while retaining their observed age, sex, educational level, and social class. The resulting individual predicted probabilities were averaged across the analytical population rather than calculated at selected covariate values. Corresponding 95% confidence intervals were calculated using the delta method.

These analyses were considered assessments of statistical heterogeneity in the observed associations and were not interpreted as evidence that lifestyle-related factors modified a causal effect of procrastination on MetS.

The interaction analyses were exploratory, and no formal adjustment for multiple testing was applied. Three multiplicative interaction tests were considered, corresponding to physical activity, Mediterranean diet adherence, and current smoking. Accordingly, interaction *p*-values were interpreted cautiously as descriptive evidence of statistical heterogeneity rather than confirmatory findings. Particular emphasis was placed on the patterns of adjusted predicted probabilities and the magnitude of differences between subgroups, rather than on statistical significance alone.

As an exploratory assessment of interaction on the additive scale, adjusted prevalence differences were calculated by subtracting the marginally standardized predicted probability of MetS in the very low/absent PPS-9 category from that in the very high PPS-9 category, separately within each physical activity and Mediterranean diet adherence subgroup. Between-subgroup differences in these prevalence differences were then calculated as descriptive additive interaction contrasts. No confidence intervals or formal hypothesis tests were calculated for the additive interaction contrasts; therefore, they were not used to establish statistical significance on the additive scale.

#### 2.9.5. Sensitivity Analyses

Several sensitivity analyses were performed to evaluate the robustness of the main findings.

First, because age distributions differed substantially across operational PPS-9 categories, the principal analyses were repeated within an age-restricted subgroup to reduce differences in age range across operational PPS-9 categories. To assess the assumption of a linear association between age and the log-odds of MetS, the fully adjusted Model 4 was additionally refitted using a cubic spline for age with four degrees of freedom instead of a single linear age term. All other covariates and the analytical sample were retained. Model fit was compared using a likelihood-ratio test, and the resulting PPS-9 odds ratios were compared with those from the original linear-age model.

Second, the analyses were repeated using ATP III and IDF definitions of MetS instead of the primary JIS definition.

Third, sex-stratified analyses were conducted to assess whether the pattern and magnitude of the observed associations and their attenuation after incorporation of lifestyle-related variables differed between men and women.

Fourth, the primary analyses were repeated after excluding participants with previously diagnosed diabetes mellitus.

Finally, an additional model incorporating BMI was examined to determine the extent to which further adjustment for general adiposity changed the estimated association between operational PPS-9 categories and MetS. This model was considered a sensitivity analysis and was not included in the primary sequence of lifestyle-adjusted models.

An additional sensitivity model was fitted for MetS including age, sex, educational level, social class, physical activity, and Mediterranean diet adherence, but excluding smoking status. This model was compared with the fully adjusted Model 4 to evaluate the sensitivity of the PPS-9–MetS association to inclusion of smoking. Marginally standardized absolute risk differences and their 95% confidence intervals were also calculated using the same analytical population and delta-method procedure described above.

To assess the sensitivity of smoking-related regression estimates to sparse-data instability, additional logistic regression models were fitted using L2 (ridge) penalization. The smoking-outcome model, adjusted for age, sex, educational level, and social class, was re-estimated across several penalty strengths (C = 100, 10, 1, and 0.1, with smaller C values indicating stronger penalization). The MetS models additionally adjusted for smoking (Models 3C and 4) were examined using the same approach. Penalized estimates were compared descriptively with the original maximum-likelihood estimates. These analyses were intended to assess sensitivity to regularization rather than to identify a definitive alternative estimate.

To explore which individual metabolic abnormalities contributed to the observed pattern of association with the composite MetS outcome, five additional exploratory analyses were conducted using each JIS component as a separate binary outcome: elevated waist circumference, elevated triglycerides, reduced HDL-C, elevated blood pressure, and elevated fasting glucose. Component status was defined using the sex-specific thresholds and other criteria described in Section 2.6. For each component, adjusted prevalence ratios and 95% confidence intervals were estimated using modified Poisson regression with a log link and robust sandwich variance estimation. All models included operational PPS-9 category, age, sex, educational level, social class, physical activity, Mediterranean diet adherence, and current smoking, corresponding to the covariate structure of Model 4. These analyses were exploratory and were not intended to estimate the causal contribution of individual components to MetS.

To assess the potential robustness of the observed association to unmeasured confounding, E-values were calculated for the adjusted prevalence ratio comparing the very high and very low/absent operational PPS-9 categories in the fully adjusted Model 4. E-values were calculated for both the point estimate and the lower bound of its 95% confidence interval using the formula E-value = PR + √[PR × (PR − 1)], applicable to prevalence ratios greater than one. This sensitivity analysis quantifies the minimum strength of association that an unmeasured confounder would need to have with both the exposure and the outcome, conditional on measured covariates, to explain away the observed association. It does not require direct measurement of the potential confounders and does not estimate their actual effects.

All statistical tests were two-sided, and statistical significance was established at *p* < 0.05. Statistical analyses were performed using SPSS Statistics version 30.0 (IBM Corp., Armonk, NY, USA).

## 3. Results

### 3.1. Characteristics of the Study Population

The final analytical sample comprised 92,184 workers. According to the operational PPS-9 categories, 18,829 participants (20.4%) were classified as very low/absent, 41,122 (44.6%) as moderate, 26,818 (29.1%) as high, and 5415 (5.9%) as very high.

Participant characteristics differed substantially across operational PPS-9 categories (Table 1). Mean age increased from 33.0 ± 6.5 years in the very low/absent category to 56.4 ± 5.7 years in the very high category. The proportion of men also increased across categories, from 46.4% in the very low/absent group to 76.1% in the very high group. The distribution of operational PPS-9 categories showed a pronounced age-related pattern. Among workers aged 18–29 years, 34.6% were classified as very low/absent, 58.8% as moderate, 6.6% as high, and fewer than 0.1% as very high. In contrast, among those aged 50–59 years, 82.0% were classified as high and 18.0% as very high, with virtually no participants in the lower categories. Among workers aged 60 years or older, 77.7% were classified as very high and 22.3% as high. These distributions indicate limited age overlap between the extreme operational PPS-9 categories, an important consideration when interpreting adjusted associations.

Marked differences were also observed in lifestyle-related characteristics (Table 1). The proportion of participants classified as physically active decreased progressively across operational PPS-9 categories, from 84.8% in the very low/absent category to 50.7% in the moderate category, 26.4% in the high category, and 9.6% in the very high category. Similarly, high adherence to the Mediterranean diet decreased from 78.3% to 48.1%, 24.4%, and 9.5%, respectively.

Anthropometric and metabolic characteristics also differed across operational PPS-9 categories (Table 1). Mean BMI increased from 22.30 ± 1.83 kg/m^2^ in the very low/absent category to 26.06 ± 4.14 kg/m^2^, 27.93 ± 4.51 kg/m^2^, and 31.01 ± 4.57 kg/m^2^ in the moderate, high, and very high categories, respectively. Mean waist circumference showed a similar pattern, increasing from 75.20 ± 7.57 cm to 82.16 ± 10.18 cm, 85.37 ± 11.38 cm, and 91.53 ± 11.17 cm, respectively.

**Figure 1:**
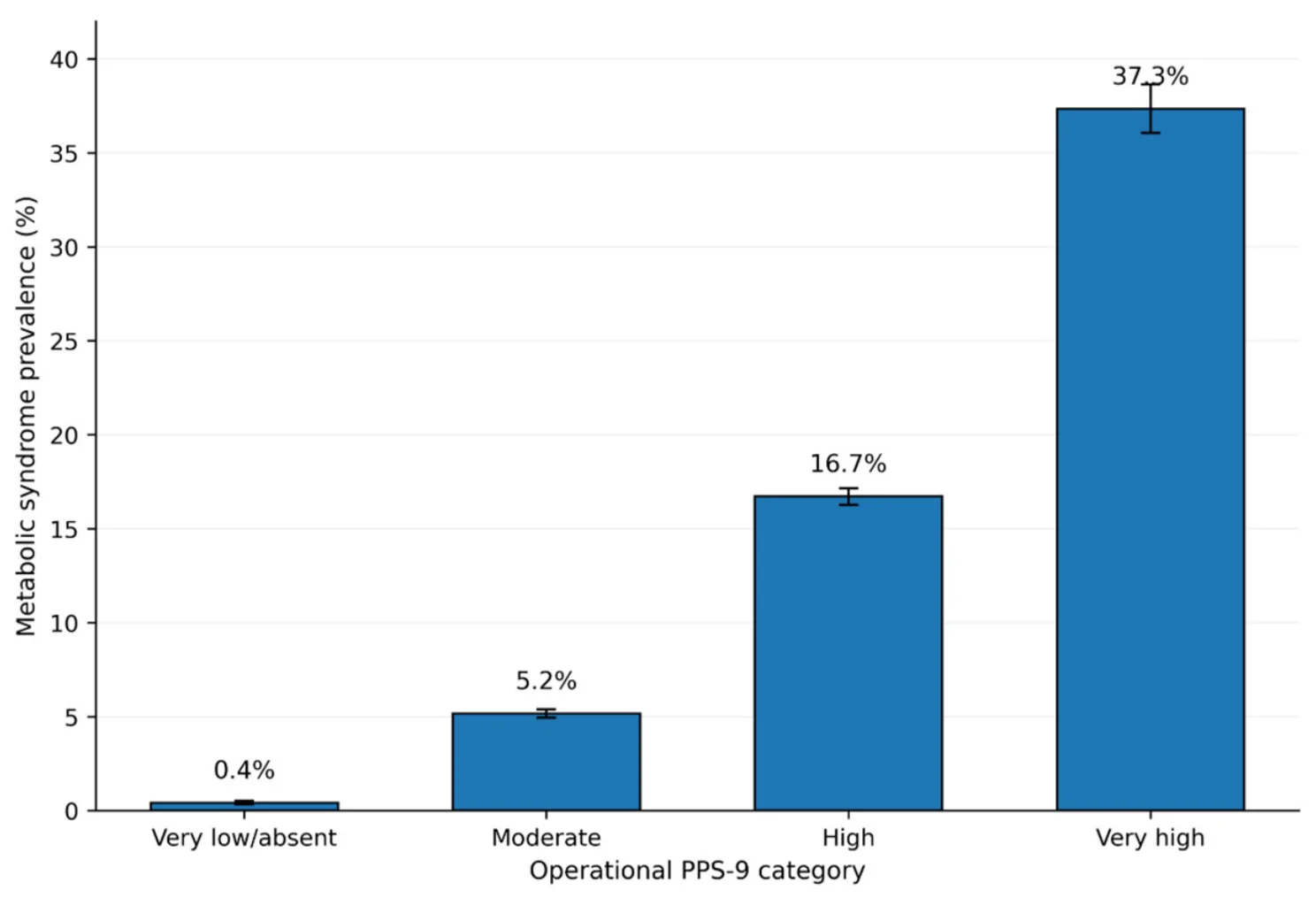
Prevalence of Metabolic Syndrome According to Operational PPS-9 Category.

The prevalence of MetS according to the JIS criteria increased across operational PPS-9 categories, from 0.4% in the very low/absent category to 5.2% in the moderate category, 16.7% in the high category, and 37.3% in the very high category (Table 1 and Figure 1).

### 3.2. Association Between Operational PPS-9 Categories and Lifestyle-Related Characteristics

Marked differences in lifestyle-related characteristics were observed across operational PPS-9 categories (Table 2). After adjustment for age, sex, educational level, and social class, progressively higher operational PPS-9 categories were associated with lower odds of being physically active. Compared with the very low/absent category, the adjusted odds ratios (aORs) for physical activity were 0.19 (95% CI 0.18–0.20) for the moderate category, 0.07 (95% CI 0.07–0.08) for the high category, and 0.02 (95% CI 0.02–0.03) for the very high category (all *p* < 0.001).

A similar pattern was observed for Mediterranean diet adherence. Compared with the very low/absent category, the adjusted odds of high Mediterranean diet adherence were lower in the moderate (aOR 0.27; 95% CI 0.26–0.28), high (aOR 0.11; 95% CI 0.10–0.11), and very high (aOR 0.04; 95% CI 0.04–0.04) operational PPS-9 categories (all *p* < 0.001) (Table 2).

Current smoking also differed markedly across operational PPS-9 categories. Current smokers represented 0.02% of participants in the very low/absent category, compared with 43.9%, 45.7%, and 42.6% in the moderate, high, and very high categories, respectively. Consequently, adjusted ORs for current smoking were very large and had wide confidence intervals: 7542.87 (95% CI 2431.60–23,398.12), 32,511.83 (95% CI 10,470.25–100,954.53), and 70,590.07 (95% CI 22,685.55–219,653.42), respectively (all *p* < 0.001) (Table 2). These estimates should be interpreted in the context of the extremely low number of current smokers in the very low/absent reference category.

Additional ridge-penalized logistic regression analyses demonstrated substantial sensitivity of the smoking-related odds ratios to the degree of penalization. In the original maximum-likelihood model, the adjusted odds ratios for current smoking were 7542.87, 32,511.83, and 70,590.07 for the moderate, high, and very high PPS-9 categories, respectively. With L2 penalization at C = 1, the corresponding estimates decreased to 836.53, 3569.16, and 7659.54. Stronger penalization (C = 0.1) reduced them further to 118.62, 473.94, and 942.13. Although the unpenalized model converged, the pronounced dependence of these estimates on penalization strength indicates substantial instability in their magnitude. In contrast, the fully adjusted MetS model was less sensitive to moderate penalization: the odds ratio for the very high PPS-9 category decreased from 30.89 to 27.95 at C = 1. These findings support cautious interpretation of the extreme smoking-related estimates.

In complementary modified Poisson regression analyses with robust variance estimation, the adjusted prevalence ratios (aPRs; 95% CIs) for current smoking were 2976.14 (959.93–9227.18), 5496.47 (1772.82–17,041.30), and 8064.37 (2600.00–25,013.10) for the moderate, high, and very high operational PPS-9 categories, respectively, compared with the very low/absent reference category. Although these estimates were substantially smaller than the corresponding odds ratios, their magnitude remained extreme because only three participants in the reference category were current smokers. The very wide confidence intervals demonstrate substantial statistical uncertainty; therefore, these prevalence ratios should not be regarded as stable estimates of the strength of association.

### 3.3. Association Between Operational PPS-9 Categories and Metabolic Syndrome

Higher operational PPS-9 categories were associated with progressively higher odds of prevalent MetS across the sequential regression models (Table 3).

In Model 1, adjusted for age and sex, the ORs for MetS were 12.74 (95% CI 10.15–15.98) for the moderate category, 62.07 (95% CI 49.34–78.08) for the high category, and 215.87 (95% CI 169.53–274.86) for the very high category, compared with the very low/absent reference category.

Additional adjustment for educational level and social class produced only minor changes in the estimates. In Model 2, the corresponding ORs were 12.63 (95% CI 10.06–15.85), 61.28 (95% CI 48.70–77.11), and 212.56 (95% CI 166.89–270.73), respectively (Table 3).

Incorporation of physical activity into Model 2 resulted in substantial statistical attenuation of the estimated association. In Model 3A, the ORs decreased to 4.47 (95% CI 3.55–5.62), 13.74 (95% CI 10.90–17.33), and 37.42 (95% CI 29.32–47.77) for the moderate, high, and very high categories, respectively.

Incorporation of Mediterranean diet adherence also resulted in statistical attenuation of the estimates. In Model 3B, the corresponding ORs were 6.00 (95% CI 4.78–7.55), 19.38 (95% CI 15.39–24.42), and 55.34 (95% CI 43.40–70.55), respectively (Table 3).

In contrast, incorporation of current smoking alone did not attenuate the estimated association. In Model 3C, the ORs were 13.99 (95% CI 11.14–17.58), 73.31 (95% CI 58.02–92.62), and 265.22 (95% CI 206.98–339.84) for the moderate, high, and very high categories, respectively. These estimates should be interpreted in the context of the highly unbalanced distribution of current smoking in the very low/absent reference category.

When physical activity, Mediterranean diet adherence, and current smoking were incorporated simultaneously (Model 4), the ORs were 4.10 (95% CI 3.26–5.17) for the moderate category, 11.67 (95% CI 9.22–14.78) for the high category, and 30.89 (95% CI 24.06–39.65) for the very high category (Table 3).

Thus, although incorporation of lifestyle-related variables was accompanied by substantial statistical attenuation of the estimated cross-sectional association, progressively higher odds of prevalent MetS remained across operational PPS-9 categories in the fully adjusted model.

In complementary modified Poisson regression analyses with robust variance estimation, the adjusted prevalence ratios (aPRs; 95% CIs) for MetS in Model 2 were 12.03 (9.60–15.07), 48.20 (38.35–60.57), and 121.09 (95.69–153.22) for the moderate, high, and very high operational PPS-9 categories, respectively, compared with the very low/absent reference category. In the fully adjusted Model 4, the corresponding aPRs were 3.95 (3.15–4.95), 9.37 (7.44–11.79), and 18.23 (14.37–23.12). These prevalence ratios were lower than the corresponding odds ratios, particularly in the higher categories, but remained unusually large and sensitive to the exceptionally low MetS prevalence in the reference group.

### 3.4. Statistical Attenuation After Incorporation of Lifestyle-Related Variables

**Figure 2:**
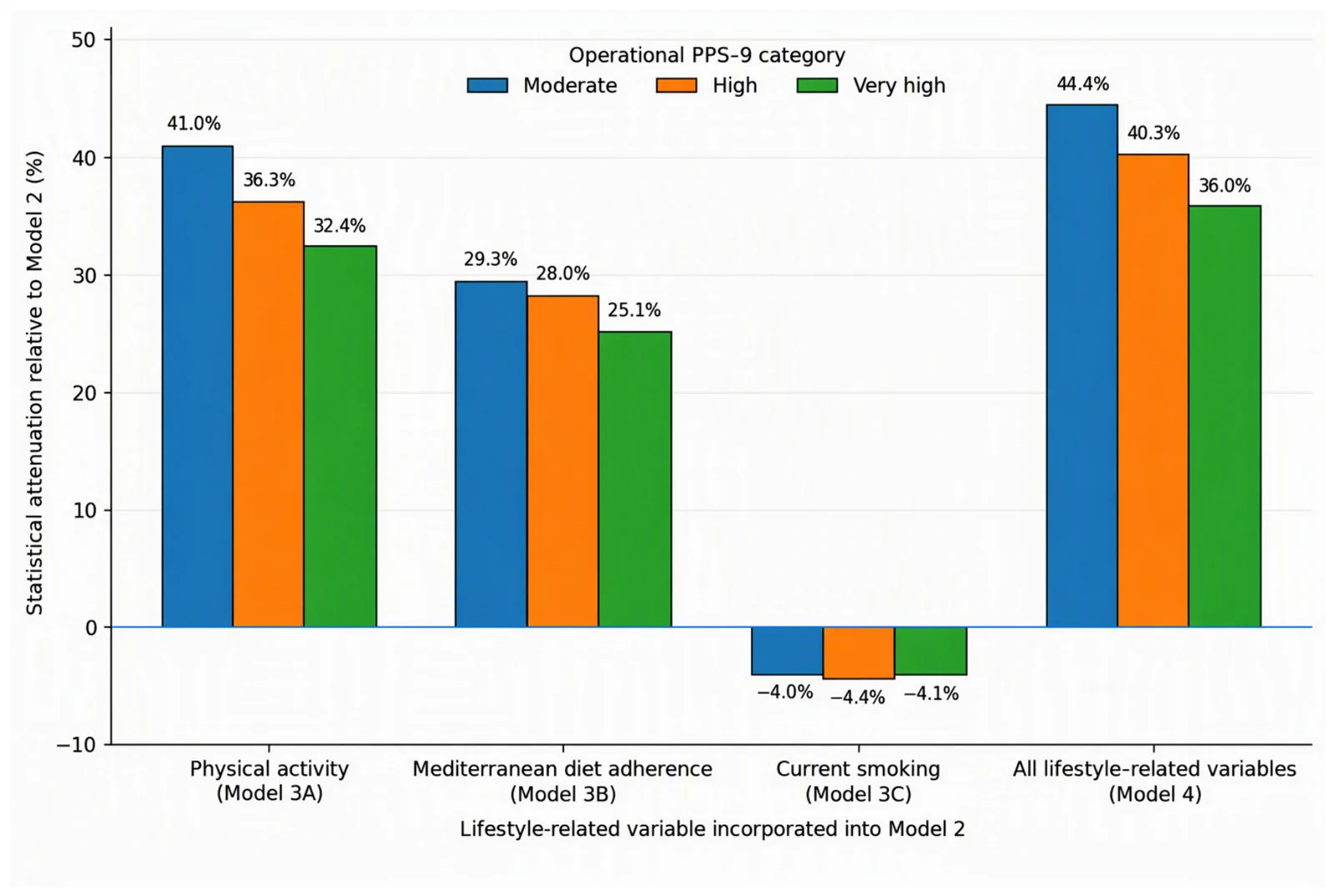
Statistical Attenuation of the Association Between Operational PPS-9 Categories and Metabolic Syndrome After Incorporation of Lifestyle-Related Variables.

The magnitude of statistical attenuation produced by incorporation of individual and combined lifestyle-related variables is presented in Table 4 and Figure 2.

Using Model 2 as the reference model, incorporation of physical activity was associated with the largest individual attenuation of the PPS-9 regression coefficients. Attenuation was 41.0% for the moderate category, 36.3% for the high category, and 32.4% for the very high category.

Incorporation of Mediterranean diet adherence was also associated with substantial attenuation, corresponding to 29.3%, 28.0%, and 25.1% for the moderate, high, and very high categories, respectively.

Incorporation of current smoking alone did not attenuate the coefficients; instead, the coefficients increased by approximately 4% across PPS-9 categories. Specifically, the calculated attenuation values were −4.0%, −4.4%, and −4.1% for the moderate, high, and very high categories, respectively.

When physical activity, Mediterranean diet adherence, and current smoking were incorporated simultaneously in Model 4, the overall attenuation relative to Model 2 was 44.4% for the moderate category, 40.3% for the high category, and 36.0% for the very high category (Table 4 and Figure 2).

Bars represent the percentage change in PPS-9 regression coefficients relative to Model 2 after incorporation of physical activity (Model 3A), Mediterranean diet adherence (Model 3B), current smoking (Model 3C), or all three lifestyle-related variables simultaneously (Model 4). Positive values indicate attenuation of the estimated cross-sectional association, whereas negative values indicate an increase in the regression coefficient. Attenuation estimates were calculated on the log-odds scale and are descriptive; they should not be interpreted as mediated proportions or causal contributions.

Abbreviations: PPS-9, 9-item Pure Procrastination Scale.

These estimates represent changes in the magnitude of the cross-sectional regression coefficients following statistical adjustment and should not be interpreted as mediated proportions or causal contributions of the lifestyle-related variables.

### 3.5. Marginally Standardized Risk Differences

To complement the comparison of logistic regression coefficients across nested models, marginally standardized probabilities of MetS were estimated for each operational PPS-9 category using the same analytical population. In Model 2, the adjusted absolute risk differences relative to the very low/absent category were 4.30, 18.65, and 43.85 percentage points for the moderate, high, and very high categories, respectively. After adjustment for physical activity (Model 3A), the corresponding differences were 3.76, 11.54, and 23.57 percentage points; after adjustment for Mediterranean diet adherence (Model 3B), they were 4.05, 12.47, and 26.02 percentage points. Adjustment for smoking alone (Model 3C) yielded differences of 4.34, 19.80, and 46.44 percentage points, respectively. In the fully adjusted model (Model 4), the differences were 3.76, 10.95, and 22.31 percentage points. The complete marginally standardized predicted probabilities, absolute risk differences, and corresponding 95% confidence intervals are presented in Supplementary Table S3.

Thus, the reduction in absolute risk differences was greater after incorporation of physical activity than after incorporation of Mediterranean diet adherence, whereas adjustment for smoking did not reduce these differences. The associations remained graded in the fully adjusted model. These results support the descriptive pattern observed in the sequential logistic models without relying exclusively on percentage changes in log-odds coefficients. They do not establish the proportion of the association explained by any lifestyle-related variable. These marginally standardized estimates provide an absolute-scale complement to the odds ratios reported in Table 3. This distinction is particularly important because the very low prevalence of MetS in the reference category and the higher prevalence in the upper PPS-9 categories make the odds ratios difficult to interpret as measures of relative prevalence. Accordingly, the magnitude of the odds ratios should not be interpreted as an equivalent multiplicative increase in MetS prevalence.

### 3.6. Interaction Analyses

Evidence of statistical interaction was observed between operational PPS-9 category and physical activity (*p* for interaction < 0.001). Adjusted predicted probabilities of MetS increased across operational PPS-9 categories in both physically inactive and active participants, although the magnitude of the association differed between activity groups (Table 5). Among physically inactive participants, the adjusted predicted probabilities of MetS were 2.46% in the very low/absent category, 9.63% in the moderate category, 24.22% in the high category, and 45.81% in the very high category. Among physically active participants, the corresponding probabilities were 0.05%, 0.25%, 1.21%, and 6.78%, respectively.

A statistically significant interaction was also observed between operational PPS-9 category and Mediterranean diet adherence (*p* for interaction < 0.001). Among participants with low Mediterranean diet adherence, the adjusted predicted probabilities of MetS were 1.63%, 8.98%, 23.69%, and 46.20% across the very low/absent, moderate, high, and very high PPS-9 categories, respectively. Among participants with high adherence, the corresponding probabilities were 0.06%, 0.30%, 1.53%, and 9.50%, respectively (Table 5). As a descriptive complement to the multiplicative interaction analyses, additive-scale contrasts were calculated from the marginally standardized predicted probabilities in Table 5. Comparing the very high with the very low/absent PPS-9 category, the adjusted prevalence difference was 43.35 percentage points among physically inactive participants and 6.73 percentage points among physically active participants, yielding a between-subgroup difference in prevalence differences of 36.62 percentage points. The corresponding prevalence differences were 44.57 percentage points among participants with low Mediterranean diet adherence and 9.44 percentage points among those with high adherence, yielding a between-subgroup contrast of 35.13 percentage points. These exploratory contrasts were derived from rounded predicted probabilities, and confidence intervals for the additive interaction contrasts were not estimated. Consequently, they should be interpreted descriptively and not as confirmatory evidence of additive interaction or causal effect modification.

The PPS-9 × smoking interaction was not interpreted because the very low/absent PPS-9 category contained only three current smokers, none of whom had MetS. This sparse outcome-by-exposure configuration undermines reliable inference for the interaction, irrespective of its nominal statistical significance. Consequently, smoking-specific interaction estimates and predicted probabilities were not presented in Table 5, and no substantive conclusions were drawn from this interaction.

Overall, these interaction analyses indicate statistical heterogeneity in the cross-sectional association between operational PPS-9 category and prevalent MetS according to physical activity and Mediterranean diet adherence. These findings should not be interpreted as evidence that lifestyle-related characteristics modify a causal effect of procrastination on MetS.

### 3.7. Sensitivity Analyses

The association between higher operational PPS-9 categories and prevalent MetS remained evident across the prespecified sensitivity analyses, although its magnitude varied according to the analytical specification and MetS definition (Table 6).

In the additional model incorporating physical activity and Mediterranean diet adherence but excluding smoking, the adjusted ORs (95% CIs) for MetS were 4.27 (3.40–5.38), 12.85 (10.19–16.21), and 34.77 (27.23–44.38) for the moderate, high, and very high PPS-9 categories, respectively. The corresponding estimates in the fully adjusted Model 4, which additionally included smoking, were 4.10 (3.26–5.17), 11.67 (9.22–14.78), and 30.89 (24.06–39.65). Marginally standardized absolute risk differences in the model excluding smoking were 3.73, 11.31, and 23.11 percentage points, compared with 3.76, 10.95, and 22.31 percentage points in Model 4, respectively. Thus, the graded PPS-9–MetS association persisted without smoking adjustment, although the estimates changed modestly when smoking was included.

When age was modeled flexibly using a cubic spline with four degrees of freedom, the fully adjusted ORs (95% CIs) for MetS were 4.17 (3.31–5.25), 14.33 (11.31–18.15), and 50.33 (39.05–64.86) for the moderate, high, and very high PPS-9 categories, respectively (Table 6). The flexible-age model showed better fit than the corresponding linear-age model (likelihood-ratio χ^2^ = 498.36; 3 degrees of freedom; *p* < 0.001). The graded association persisted, although the estimated magnitude increased, particularly in the very high category.

When MetS was defined according to ATP III criteria, the fully adjusted ORs were 2.86 (95% CI 2.23–3.67) for the moderate category, 7.31 (95% CI 5.67–9.43) for the high category, and 15.78 (95% CI 12.05–20.66) for the very high category. Using the IDF definition, the corresponding ORs were 17.00 (95% CI 10.51–27.48), 66.18 (95% CI 40.80–107.34), and 248,36 (95% CI 151.88–406.14), respectively.

Restriction of the analysis to participants aged 40–49 years included 27,153 workers, distributed across the very low/absent (*n* = 3618), moderate (*n* = 12,572), high (*n* = 10,261), and very high (*n* = 702) operational PPS-9 categories. The corresponding numbers of MetS cases were 30, 882, 1933, and 396, respectively, across these four categories.

The corresponding adjusted ORs for MetS were 4.16 (95% CI 2.86–6.04), 14.84 (95% CI 10.14–21.72), and 65.19 (95% CI 42.94–98.98) for the moderate, high, and very high categories, respectively, compared with the very low/absent reference category (Table 6). Although all four categories were represented within this narrower age range, their sample sizes remained unequal, particularly for the very high category. Thus, age restriction reduced the age range examined but did not establish full comparability between exposure groups.

Sex-stratified analyses showed a similar graded pattern in both men and women. Among men, the corresponding ORs were 4.76 (95% CI 3.44–6.58), 12.81 (95% CI 9.22–17.79), and 32.59 (95% CI 23.15–45.87), whereas among women they were 3.64 (95% CI 2.61–5.09), 12.28 (95% CI 8.65–17.43), and 37.66 (95% CI 25.66–55.27).

Exclusion of participants with diagnosed diabetes produced estimates similar to those of the primary fully adjusted model, with ORs of 4.23 (95% CI 3.35–5.35), 11.83 (95% CI 9.30–15.04), and 29.89 (95% CI 23.18–38.55), respectively.

Additional adjustment for BMI resulted in further statistical attenuation of the estimated cross-sectional association. Nevertheless, progressively higher odds of prevalent MetS remained across operational PPS-9 categories, with ORs of 1.94 (95% CI 1.53–2.45), 3.65 (95% CI 2.85–4.66), and 5.96 (95% CI 4.57–7.77) for the moderate, high, and very high categories, respectively (Table 6).

Overall, the graded cross-sectional association between operational PPS-9 category and prevalent MetS was observed across the sensitivity analyses, while the magnitude of the estimates varied according to the MetS definition and additional covariate adjustment.

To further assess the influence of the diagnostic definition of metabolic syndrome, the number and prevalence of MetS cases across PPS-9 categories were calculated using the JIS, ATP III, and IDF criteria, as presented in Table 7.

The prevalence of MetS increased progressively across PPS-9 categories under all three diagnostic definitions (Table 7). Overall, MetS prevalence was 9.44% according to JIS, 6.91% according to ATP III, and 7.60% according to the recalculated IDF definition. In the very low/absent PPS-9 category, the corresponding prevalences were 0.41%, 0.36%, and 0.09%, respectively. Although the direction of the association was consistent across definitions, the magnitude of the adjusted odds ratios differed substantially, particularly for IDF-defined MetS (Table 6).

Additional exploratory analyses of the five individual JIS components revealed heterogeneous associations with operational PPS-9 categories. After multivariable adjustment, elevated waist circumference showed the strongest positive gradient, followed by elevated blood pressure and fasting glucose. In contrast, adjusted prevalence ratios for elevated triglycerides and reduced HDL-C were below unity, despite their increasing crude prevalences across PPS-9 categories. These findings should be interpreted cautiously given the substantial differences in age distribution between PPS-9 categories and the exploratory nature of the analyses (Supplementary Table S4).

## 4. Discussion

### 4.1. Principal Findings

In this large occupational study of 92,184 Spanish workers, progressively higher operational PPS-9 categories were associated with a markedly less favorable lifestyle profile and higher prevalence of MetS. Higher PPS-9 categories were strongly associated with lower physical activity and lower adherence to the Mediterranean diet, while current smoking also differed substantially across categories. A clear graded cross-sectional association was observed between operational PPS-9 category and prevalent MetS, which remained evident after adjustment for sociodemographic and lifestyle-related characteristics.

A central finding of the present study was the substantial statistical attenuation observed after incorporation of lifestyle-related variables into the multivariable models. Physical activity was associated with the largest individual attenuation of the PPS-9 coefficients, followed by Mediterranean diet adherence. When physical activity, Mediterranean diet adherence, and current smoking were incorporated simultaneously, attenuation ranged from approximately 36% to 44% across PPS-9 categories. Nevertheless, progressively higher odds of prevalent MetS remained in the fully adjusted model. These findings indicate substantial statistical overlap between procrastination, lifestyle-related characteristics, and metabolic status, but should not be interpreted as evidence of mediation or of a causal pathway.

The interaction analyses provided additional evidence of heterogeneity in these cross-sectional associations. The association between operational PPS-9 category and MetS differed according to physical activity and Mediterranean diet adherence, with substantially lower adjusted probabilities of MetS among physically active participants and those with high Mediterranean diet adherence across PPS-9 categories. The interaction with smoking also reached statistical significance, although the extreme sparsity of current smokers in the very low/absent PPS-9 category precluded reliable substantive interpretation of the corresponding estimates.

Finally, the graded association between operational PPS-9 category and MetS was consistently observed across the prespecified sensitivity analyses, including alternative MetS definitions, age-restricted and sex-stratified analyses, and exclusion of participants with diagnosed diabetes. Additional adjustment for BMI produced further statistical attenuation, although higher operational PPS-9 categories remained associated with higher odds of prevalent MetS.

### 4.2. Comparison with Previous Literature

The present findings are consistent with an expanding body of evidence linking procrastination with less favorable health-related characteristics. A recent systematic review and three-level meta-analysis including 66 studies and 32,131 participants reported a moderate association between procrastination and adverse health outcomes [26]. This broader evidence supports the relevance of procrastination beyond academic performance or productivity and is consistent with the marked differences in lifestyle and metabolic characteristics observed across operational PPS-9 categories in the present occupational population.

The association observed with physical activity is particularly consistent with recent evidence. A systematic review and meta-analysis of 43 studies reported an inverse association between physical activity and procrastination, with higher levels of physical activity associated with lower procrastination [27]. Our findings extend these observations to a large occupational population, showing a pronounced graded association between operational PPS-9 category and physical activity. Moreover, incorporation of physical activity into the multivariable models was accompanied by the largest individual statistical attenuation of the PPS-9–MetS coefficients.

The findings regarding Mediterranean diet adherence provide an additional behavioral dimension. Evidence specifically examining procrastination in relation to Mediterranean dietary patterns remains limited, whereas the association between Mediterranean diet adherence and metabolic health is well established. A large meta-analysis including 50 studies and more than 500,000 participants reported that greater adherence to a Mediterranean dietary pattern was associated with a more favorable profile across several components of MetS [28]. Similarly, a meta-analysis of observational studies including 33,847 participants found an inverse association between Mediterranean diet adherence and MetS occurrence [29]. In the present study, progressively higher operational PPS-9 categories were associated with markedly lower Mediterranean diet adherence, and incorporation of diet adherence into the regression models was accompanied by substantial statistical attenuation of the PPS-9–MetS association. These findings identify Mediterranean diet adherence as an important behavioral characteristic that statistically overlaps with both procrastination and metabolic status, without establishing the temporal direction of these relationships.

The present findings should be distinguished from our three earlier publications based on exactly the same 92,184 workers and underlying occupational health dataset [8,9,10]. The initial investigation [8] had already documented the graded cross-sectional association between operational PPS-9 categories and MetS, including its persistence after multivariable adjustment. Therefore, neither the association itself nor its replication in this manuscript constitutes a new finding. The subsequent studies examined different cardiometabolic outcomes: insulin resistance and visceral adiposity phenotypes [9] and heart age acceleration [10]. Although the insulin resistance and adiposity study also reported attenuation after adjustment for demographic and lifestyle-related characteristics, it did not address the specific contribution of individual lifestyle variables to the procrastination–MetS association. The additional contribution of the present analysis is the separate comparison of statistical attenuation after incorporating physical activity, Mediterranean diet adherence, and smoking; the evaluation of interactions between operational PPS-9 categories and lifestyle characteristics; and the presentation of marginally standardized MetS prevalences and absolute prevalence differences. Physical activity showed the greatest individual attenuation, followed by Mediterranean diet adherence, while the interaction analyses identified heterogeneity according to these lifestyle characteristics. These results refine the interpretation of an association already reported in this population rather than providing independent confirmation of it. Together with recent meta-analytic evidence linking procrastination with adverse health outcomes [26], they reinforce the relevance of examining lifestyle-related correlates of procrastination. Given the complete participant overlap and cross-sectional design, the findings should be interpreted as descriptive statistical associations, without implying mediation, temporality, or causality.

### 4.3. Potential Mechanisms

Several behavioral and psychosocial processes described in previous literature may be compatible with the associations observed in the present study, although the cross-sectional design does not allow these processes to be established as mechanisms or causal pathways. Procrastination has been conceptualized as a form of self-regulatory failure in which short-term mood regulation may take priority over actions whose benefits are delayed [30]. From this perspective, postponement may coexist with difficulties initiating or maintaining behaviors that require sustained self-regulation, including regular physical activity and adherence to healthy dietary patterns. This interpretation is consistent with the pronounced gradients in physical activity and Mediterranean diet adherence observed across operational PPS-9 categories in our population.

Stress may represent another relevant correlate. Procrastination and psychological stress are consistently associated across different populations and contexts, and their relationship may be dynamic and potentially bidirectional [31]. Recent meta-analytic evidence has also shown an association between procrastination and poorer self-rated health, with the magnitude of this association reduced after accounting for perceived stress [32]. Although psychological stress was not available in the present dataset and therefore could not be examined directly, these findings suggest that stress-related characteristics may coexist with procrastination and health status and represent an important source of residual confounding in the observed PPS-9–MetS association.

The substantial statistical attenuation observed after incorporation of physical activity and Mediterranean diet adherence further indicates that behavioral characteristics overlap considerably with the PPS-9–MetS association. Physical inactivity and unfavorable dietary patterns are themselves associated with adiposity, insulin resistance, dyslipidemia, elevated blood pressure, and other metabolic abnormalities relevant to MetS [33]. Consistent with this overlap, additional adjustment for BMI in our sensitivity analysis produced further marked attenuation of the association. However, neither the sequential adjustment models nor the BMI sensitivity analysis establishes temporal ordering among procrastination, lifestyle behaviors, adiposity, and MetS. These characteristics may develop concurrently, influence one another over time, or reflect other unmeasured behavioral, psychosocial, occupational, or clinical factors.

Accordingly, the present findings are compatible with a multifactorial interpretation in which behavioral procrastination, lifestyle characteristics, adiposity, and metabolic abnormalities coexist within a broader health-related profile.

### 4.4. Clinical and Public Health Implications

The present findings may have relevance for preventive medicine and occupational health because higher operational PPS-9 categories identified groups of workers with markedly less favorable lifestyle and metabolic profiles. The coexistence of higher procrastination levels, lower physical activity, lower Mediterranean diet adherence, greater adiposity, and higher prevalence of MetS suggests that procrastination assessment may provide complementary information when characterizing behavioral profiles associated with cardiometabolic health.

The occupational setting may be particularly relevant for these observations. Workplace health assessments provide opportunities for the simultaneous identification of behavioral and cardiometabolic characteristics in large populations, and previous research in working populations has supported the usefulness of occupational health settings for cardiometabolic screening and lifestyle-oriented prevention [34,35]. In this context, the strong associations observed between operational PPS-9 categories, physical activity, Mediterranean diet adherence, and MetS suggest that behavioral self-regulation characteristics could be considered alongside established lifestyle factors when describing workers with less favorable health profiles.

### 4.5. Strengths and Limitations

The present study has several important strengths. First, the very large analytical sample of 92,184 workers provided substantial statistical precision and allowed the association between operational PPS-9 categories, lifestyle-related characteristics, and MetS to be examined across multiple analytical approaches. The multicenter occupational setting also provided a large real-world population undergoing standardized preventive health assessments. Anthropometric measurements, blood pressure, and fasting biochemical parameters were obtained by trained personnel using standardized procedures and certified laboratories, reducing dependence on self-reported information for the definition of the primary metabolic outcome. MetS was primarily defined according to the harmonized Joint Interim Statement criteria, and the availability of alternative ATP III and IDF classifications allowed the robustness of the findings to be evaluated across different established definitions of the syndrome.

A further strength is the standardized assessment of lifestyle-related characteristics using the IPAQ-SF, MEDAS, and occupational health questionnaires. The sequential modeling strategy allowed physical activity, Mediterranean diet adherence, and smoking to be examined separately and jointly, while changes in regression coefficients were interpreted descriptively rather than as evidence of mediation.

The analyses also addressed several potential sources of uncertainty. Age, sex, educational level, and social class were incorporated into the adjustment strategy, which is particularly relevant given the marked demographic differences across PPS-9 categories. Interaction analyses complemented the main models by examining statistical heterogeneity according to physical activity, Mediterranean diet adherence, and smoking status, and adjusted predicted probabilities facilitated interpretation of significant interactions without assigning them a causal meaning. In addition, an extensive set of sensitivity analyses examined an age-restricted population, alternative ATP III and IDF definitions of MetS, sex-specific associations, exclusion of participants with previously diagnosed diabetes, and additional adjustment for BMI. Although the graded association persisted across these analytical specifications, the substantial age differences between PPS-9 categories warrant particular caution. The age-restricted analysis included participants from all four categories but did not establish full comparability between groups. Furthermore, flexible adjustment for age using a cubic spline improved model fit and yielded larger odds ratios, particularly in the very high PPS-9 category, compared with linear age adjustment (Table 6). Thus, the direction and graded pattern of the association were maintained, but its estimated magnitude was sensitive to the functional form of age adjustment. Residual confounding related to the pronounced age gradient cannot be excluded.

Cognitive functioning may provide an additional perspective on the relationship between behavioral procrastination and cardiometabolic health. Executive functions, including planning, inhibitory control, and cognitive flexibility, are conceptually relevant to behavioral self-regulation and the initiation and completion of intended actions. Age-related differences in cognitive functioning may therefore be particularly relevant when interpreting the pronounced age gradient observed across operational PPS-9 categories. In a study of older adults with arterial hypertension, Kapusta et al. reported an association between increasing age and lower cognitive performance, although hypertension duration was not significantly associated with Mini-Mental State Examination scores [36]. While that study did not investigate procrastination or specifically assess executive functions, it highlights the importance of considering cognitive functioning alongside age and cardiovascular health. Cognitive performance and executive functions were not assessed in our dataset; consequently, their potential contribution to the observed associations remains hypothetical and warrants investigation in future studies.

Although the association between procrastination and MetS showed a consistent graded pattern across diagnostic definitions, its magnitude varied considerably. For the very high PPS-9 category, the fully adjusted OR was 15.78 under ATP III, 30.89 under JIS, and 248.36 under the recalculated IDF definition. These differences may partly reflect the distinct waist circumference thresholds and the requirement for central obesity under IDF, which affect case classification. In particular, the very low prevalence of IDF-defined MetS in the reference category (0.09%) may contribute to the large relative estimates. Therefore, consistency in the direction of association should not be interpreted as stability in its magnitude.

An additional limitation concerns the absence of information on medication use for metabolic abnormalities. MetS classification was based exclusively on measured anthropometric, blood pressure, and biochemical parameters, without incorporating pharmacological treatment for hypertension, dyslipidemia, or hyperglycemia. Consequently, participants receiving effective treatment whose measured values were below the diagnostic thresholds may have been misclassified as not meeting the corresponding MetS components. This could have resulted in an underestimation of the prevalence of MetS and may have influenced the magnitude of the observed associations. The direction and extent of any resulting bias in the association estimates cannot be determined from the available data.

Several limitations should nevertheless be considered. The most important is the cross-sectional design. Procrastination, lifestyle characteristics, adiposity, and metabolic abnormalities were assessed within the same observational framework, and their temporal ordering cannot therefore be established. Consequently, the observed attenuation after incorporation of lifestyle-related variables cannot be interpreted as mediation, and the interaction analyses cannot establish that lifestyle behaviors modify a causal effect of procrastination on MetS. Reverse and bidirectional relationships are also possible. For example, less favorable metabolic health, obesity, fatigue, reduced physical functioning, or other health-related characteristics could coexist with or precede differences in procrastination and lifestyle behaviors. The present analyses therefore characterize cross-sectional associations and statistical overlap only. This limitation was explicitly incorporated into the prespecified analytical framework of the study.

Reverse causation deserves particular consideration. Workers with established metabolic abnormalities, obesity, diabetes, or other chronic health conditions may experience fatigue, reduced physical functioning, sleep disturbances, psychological distress, or increased demands associated with medical appointments and disease management. These circumstances could influence the initiation and completion of everyday tasks, thereby affecting self-reported procrastination and responses to PPS-9 items. Similarly, a diagnosis of metabolic disease may prompt changes in physical activity, dietary practices, or smoking behavior, whereas disease-related symptoms may restrict activity or interfere with the maintenance of healthy routines. Thus, prevalent metabolic disease could influence both the behavioral exposure and the lifestyle-related covariates, rather than necessarily occurring downstream of them. Consequently, the percentages of attenuation observed after lifestyle adjustment cannot be interpreted as the proportion of the association explained by these behaviors. Nor does attenuation establish the direction of the underlying relationships: changes in model coefficients may reflect shared correlates, reverse or bidirectional associations, residual confounding, and statistical properties of the models. Conversely, the persistence of an association after adjustment does not establish an independent causal effect of procrastination on MetS.

Second, behavioral procrastination was assessed using a nine-item Spanish-language translation of the PPS-9, prepared for occupational health examinations with the participation of a psychologist and another professional. Although professional expertise was incorporated into the translation process, this version has not undergone formal psychometric validation in the study population. Therefore, its internal consistency, factorial structure, and measurement equivalence with the original instrument remain unestablished. Moreover, the analytical dataset retained only the final operational PPS-9 category, without individual item responses or continuous total scores. Consequently, retrospective assessments of reliability and factorial validity, as well as analyses using continuous PPS-9 scores, were not possible. The four categories used in this study represent operational groupings rather than clinically validated, diagnostic, or prognostic thresholds. Categorization also reduces the information contained in a continuous measure and may create apparently discrete differences within an underlying continuum. These limitations introduce uncertainty regarding exposure measurement and potential misclassification. Accordingly, associations between operational PPS-9 categories should not be interpreted as evidence of established clinical or biological thresholds. An additional methodological consideration is the non-collapsibility of odds ratios in logistic regression. Changes in log-odds coefficients across nested models may partly reflect differences in model scaling rather than substantive changes attributable to the newly included covariates. Calculating percentage attenuation on the log-odds scale does not eliminate this limitation. We therefore complemented the coefficient-based comparisons with marginally standardized predicted probabilities and absolute risk differences, evaluated over the same analytical population. The broadly consistent descriptive pattern on the absolute probability scale strengthens the interpretation that physical activity and Mediterranean diet adherence are associated with differences in the observed PPS-9–MetS relationship. Nevertheless, comparisons across differently adjusted models do not quantify causal contributions, mediated proportions, or the amount of association explained by these behaviors. Furthermore, standardized predictions may be sensitive to limited covariate overlap, particularly given the sparse number of smokers in the lowest PPS-9 category.

Third, procrastination and the principal lifestyle-related characteristics were based wholly or partly on self-reported questionnaire information. Physical activity assessed with IPAQ-SF may be affected by recall error and social desirability, and dichotomization at 600 MET-min/week simplifies variation in the frequency, duration, intensity, and type of activity. Similarly, dichotomizing MEDAS scores at ≥9 versus <9 does not capture the full range of dietary adherence and does not provide detailed quantitative information on energy intake, nutrient composition, individual food consumption, or changes in diet over time. Consequently, some degree of exposure misclassification is possible. Objective measures such as accelerometry, repeated dietary assessment, or longitudinal behavioral monitoring were not available and could provide more detailed characterization in future research. Furthermore, the available analytical dataset retained only the dichotomized classifications for Mediterranean diet adherence (high vs. low) and physical activity (active vs. inactive), rather than the original continuous MEDAS scores or detailed IPAQ-SF-derived activity measures. Consequently, sensitivity analyses using the original MEDAS score or more granular physical activity measures could not be performed. Together with the absence of continuous PPS-9 scores, these data limitations prevented an assessment of whether the observed associations were sensitive to the selected categorization thresholds. Importantly, the larger descriptive reduction in the PPS-9–MetS association observed after adjustment for physical activity compared with Mediterranean diet adherence should not be interpreted as evidence that physical activity is intrinsically more important than diet. The comparison depends on the measurement instruments, dichotomization thresholds, exposure distributions, and potential measurement error associated with each behavior. Therefore, the observed differences between models describe patterns of statistical adjustment within this dataset and do not provide direct estimates of the relative importance or causal contributions of physical activity and Mediterranean diet adherence.

An additional limitation concerns the unusually strong relationship between age and the operational PPS-9 categories. Mean age increased markedly across categories, a pattern that contrasts with much of the previous literature reporting greater procrastination among younger adults. The near absence of lower procrastination categories among older workers also indicates limited age overlap, particularly at the extremes of the distribution. Although age was included in the multivariable models and a flexible cubic-spline specification was examined in sensitivity analyses (Table 6), statistical adjustment cannot fully overcome the lack of comparable participants across age strata. Consequently, residual confounding by age and potential limitations in the interpretation of the operational PPS-9 categories cannot be excluded. This unusual age pattern warrants further investigation using item-level questionnaire data and independently validated procrastination measures.

Fourth, smoking deserves particular caution. The distribution of current smoking across operational PPS-9 categories was extremely unbalanced, with only three current smokers in the very low/absent reference category. This sparse reference cell generated exceptionally large and imprecise smoking-related odds ratios and limits substantive interpretation of the association between PPS-9 category and smoking. It also makes the PPS-9 × smoking interaction considerably less stable than the corresponding physical activity and dietary interactions. The smoking findings should therefore be interpreted primarily as evidence of an unusual and highly asymmetric distribution within this dataset rather than as precise estimates of the magnitude of association. Importantly, this limitation does not apply to the physical activity and Mediterranean diet analyses to the same extent. The smoking distribution is an observed characteristic of the dataset and should not be interpreted as evidence of a general population pattern. Internal checks of the available analytical dataset confirmed the reported smoking frequencies and consistency between the retained smoking-status variable and its binary recoding. However, because the original source records, questionnaire routing information, and pre-extraction linkage records were not available for retrospective review, potential errors arising during initial data collection, extraction, or record linkage could not be independently excluded. The exceptionally low prevalence of current smoking in the very low/absent PPS-9 category therefore remains an unresolved data-quality concern. Consequently, smoking-related estimates, particularly those relying on this sparse reference category, should be interpreted with substantial caution. The very low prevalence of MetS in the same reference category (78 cases among 18,829 participants; 0.4%) also warrants caution, because all category-specific MetS odds ratios are estimated relative to this group. Although the analytical data support the reported case count, the absence of original source records prevents independent verification of the initial extraction and coding procedures.

The additional penalized regression analyses further demonstrated that the magnitude of the smoking-related odds ratios was highly sensitive to regularization. Although the conventional logistic models converged, convergence alone does not exclude sparse-data bias or establish reliable estimation. The penalized analyses indicate that the exceptionally large smoking-related odds ratios are not robust in magnitude. Accordingly, strong substantive interpretations of the smoking-related associations are not warranted. The additional MetS model incorporating physical activity and Mediterranean diet adherence but excluding smoking showed that the graded association across operational PPS-9 categories persisted without smoking adjustment (Table 6). Although the estimates differed modestly from those of the fully adjusted Model 4, these differences should not be interpreted causally. Given the sparse smoking data and the instability of smoking-related estimates, the nominally significant PPS-9 × smoking interaction was not considered reliable evidence of effect modification.

Fifth, residual and unmeasured confounding cannot be excluded. Although the models incorporated major demographic, socioeconomic, and lifestyle-related characteristics, information on several potentially relevant variables was not systematically available. In particular, diagnosed depression, anxiety disorders, attention-deficit/hyperactivity disorder, and psychotropic medication use could not be included. Other potentially relevant characteristics not comprehensively assessed in the present analyses include psychological distress, perceived stress, sleep duration and quality, insomnia, chronotype, alcohol consumption, occupational stress, shift work, working hours, job strain, burnout, income, marital or family circumstances, caregiving responsibilities, personality characteristics, executive functioning, medication use, and other chronic conditions. Some of these characteristics could be associated with procrastination, lifestyle behaviors, and metabolic health simultaneously. Therefore, the adjusted estimates should not be interpreted as representing associations independent of every relevant behavioral, psychological, occupational, or clinical characteristic. To quantify sensitivity to unmeasured confounding, we calculated E-values based on the fully adjusted prevalence ratio for the very high versus very low/absent PPS-9 category. The point-estimate E-value was 35.95, and the E-value for the lower 95% confidence limit was 28.23. Under the assumptions of the E-value framework, these values indicate the minimum strength of association, on the prevalence-ratio scale, that an unmeasured confounder would need to have with both PPS-9 category and MetS, conditional on the measured covariates, to explain away the observed association or move its confidence interval to include the null. However, these values do not exclude residual confounding, address the limited age overlap between PPS-9 categories, or establish causality. In particular, the exceptionally low MetS prevalence in the reference category warrants caution when interpreting these sensitivity estimates.

Sixth, although social class and educational level were incorporated as indicators of socioeconomic position, socioeconomic and occupational circumstances were necessarily simplified. Occupational activity according to CNAE was available but was not included in the primary models because social class was selected as a more parsimonious indicator. This approach reduces model complexity but cannot fully characterize potential differences associated with specific industries, occupational exposures, physical job demands, employment conditions, shift schedules, or workplace environments. Residual socioeconomic and occupational confounding therefore remains possible.

Seventh, the analytical treatment of lifestyle variables necessarily involved simplification. Physical activity and Mediterranean diet adherence were analyzed as binary characteristics, and current smoking was used as the principal smoking-related variable. These classifications facilitated interpretation and comparison across models but may obscure dose–response relationships within each behavior. In particular, the analyses do not distinguish the metabolic relevance of different amounts or types of physical activity, different degrees of Mediterranean diet adherence, or smoking intensity and duration. Nor were composite lifestyle scores examined. Consequently, the reported attenuation estimates describe the statistical overlap associated with the operational definitions used in this study rather than the complete behavioral variability present in the population.

Eighth, MetS is a composite categorical outcome comprising five metabolic abnormalities that may differ in their associations with operational PPS-9 categories. To investigate this heterogeneity, we conducted additional exploratory analyses of the individual JIS components. The adjusted associations were not uniform: elevated waist circumference showed the strongest positive gradient across PPS-9 categories, whereas elevated blood pressure and fasting glucose showed more moderate positive gradients. In contrast, the adjusted prevalence ratios for elevated triglycerides and reduced HDL-C were below unity despite increasing crude prevalences across PPS-9 categories. These divergent patterns should be interpreted cautiously, particularly given the pronounced differences in age distribution and limited overlap between PPS-9 categories. Component-specific associations do not quantify the relative contribution of each abnormality to the composite MetS association, nor do they establish protective or causal effects. Furthermore, the analyses do not characterize the joint distribution or severity of metabolic abnormalities within individuals. Replication of the overall MetS association using ATP III and IDF criteria supports consistency across outcome definitions but does not eliminate these interpretative limitations.

Ninth, BMI adjustment represents a particular analytical challenge because adiposity is closely related to the metabolic phenotype used to define MetS. Although BMI is not itself a component of the JIS definition, waist circumference is one of its five diagnostic components, and BMI is strongly correlated with central adiposity and several other metabolic abnormalities. Consequently, adjustment for BMI does not simply provide additional control for an independent confounder; it changes the association being estimated by conditioning on a variable closely related to the outcome definition. The BMI-adjusted odds ratios of 1.94, 3.65, and 5.96 for the moderate, high, and very high PPS-9 categories, respectively, should therefore be interpreted as associations conditional on BMI rather than as directly comparable estimates of the same association assessed in the primary models. Their attenuation may reflect statistical overlap with adiposity, but it cannot establish mediation, identify a direct effect independent of adiposity, or determine whether adiposity precedes or follows procrastination-related behaviors. For these reasons, BMI adjustment was retained exclusively as a sensitivity analysis and was not incorporated into the primary sequential models.

Tenth, the use of logistic regression warrants consideration because MetS was common in the higher PPS-9 categories. Under these circumstances, odds ratios can be numerically larger than corresponding prevalence ratios and should not be interpreted as risk ratios. The reported ORs therefore quantify cross-sectional odds of prevalent MetS rather than prospective disease risk. This distinction is particularly important when communicating the large estimates observed for the higher operational PPS-9 categories.

Eleventh, participants with incomplete information required for the analyses were excluded. If missingness was associated with procrastination, lifestyle behaviors, or metabolic status, complete-case analysis could introduce selection bias. The available study data do not establish whether excluded individuals differed systematically from those retained in the analytical sample. Accordingly, some influence of missing-data-related selection cannot be excluded.

Finally, external validity is necessarily limited by the occupational nature of the population. Participants were active Spanish workers aged 18–69 years undergoing routine occupational health examinations. The findings may therefore not be directly generalizable to unemployed individuals, retired populations, people unable to work because of illness, adolescents, substantially older adults, or populations from different cultural, occupational, socioeconomic, or healthcare settings. A healthy-worker effect may have influenced the observed gradients because inclusion was restricted to individuals who remained in active employment and attended occupational health examinations. Workers with more severe metabolic disease, functional limitations, or difficulties maintaining employment may have been less likely to enter or remain in the analytical population. Such selection could alter the distribution of both PPS-9 categories and MetS, potentially attenuating or accentuating their observed association; the direction and magnitude of this bias cannot be determined from the available data. Job tenure may also be relevant: longer employment duration could be associated with age, cumulative occupational exposures, work routines, health-related selection, and opportunities to develop or modify behavioral patterns. However, individual job tenure and employment histories were not available for analysis, so these possibilities could not be evaluated directly. Adjustment for age and occupational social class cannot fully account for these selection processes or for differences in employment duration. Conversely, the large and heterogeneous occupational sample provides valuable evidence from a real-world working population, but replication in independent population-based and international cohorts will be necessary to establish the broader generalizability of the observed associations.

An additional limitation concerns the complete-case analysis, which excluded 2143 participants (2.3%) because of missing data. Individual-level records for these excluded participants were not available in the retained analytical dataset. Consequently, their characteristics could not be compared with those of included participants, and multiple imputation could not be performed. Although the proportion excluded was relatively small, differences between included and excluded participants cannot be ruled out, and potential selection bias due to missing data remains a source of uncertainty.

### 4.6. Future Directions

Future research should primarily adopt longitudinal designs with repeated assessments of procrastination, lifestyle behaviors, adiposity, and metabolic health. Such studies would allow the temporal ordering of these characteristics to be examined and would help determine whether changes in procrastination precede, follow, or occur concurrently with changes in physical activity, dietary habits, body composition, and metabolic abnormalities. This is particularly important because the present cross-sectional analyses quantify associations and statistical attenuation but cannot distinguish among these temporal possibilities.

Future studies should also improve the measurement of both procrastination and lifestyle-related characteristics. Validation of the specific Spanish PPS-9 adaptation used in this occupational population, including assessment of its reliability, factor structure, measurement invariance, and longitudinal stability, would strengthen interpretation of subsequent findings. The operational PPS-9 categories used here should also be examined against continuous scores and independently replicated before any clinical or prognostic thresholds are proposed. Objective physical activity assessment using accelerometry or wearable devices, repeated dietary assessment, and more detailed characterization of smoking history and intensity would additionally reduce measurement error and allow evaluation of dose–response patterns that could not be fully explored using the dichotomous lifestyle classifications applied in the present study.

A broader assessment of psychological, behavioral, and occupational characteristics is also warranted. Future datasets should ideally incorporate perceived stress, depression, anxiety, ADHD symptoms, sleep duration and quality, chronotype, alcohol consumption, executive functioning, personality characteristics, occupational stress, shift work, working hours, burnout, medication use, and other potentially relevant clinical and socioeconomic factors. Psychiatric diagnoses and psychotropic medication use were not systematically available in the present dataset, limiting the extent to which these characteristics could be considered in the adjusted analyses. Incorporating these variables would permit a more comprehensive characterization of the behavioral and psychosocial profiles that coexist with procrastination and metabolic health.

Further work should also examine individual components and continuous markers of metabolic health rather than relying exclusively on the binary presence of MetS. Although the consistency of the present findings across JIS, ATP III, and IDF definitions strengthens their robustness, longitudinal analyses of waist circumference, blood pressure, glucose regulation, triglycerides, HDL-C, insulin resistance markers, and measures of visceral adiposity could clarify whether particular metabolic characteristics show stronger or earlier associations with procrastination-related behavioral profiles.

The observed statistical interactions with physical activity and Mediterranean diet adherence also merit replication in independent populations. Future studies should evaluate these associations using continuous measures of activity and dietary adherence and determine whether similar patterns are observed across age groups, sexes, occupational sectors, socioeconomic strata, and different cultural settings. The smoking findings require particular confirmation in independent samples because of the extreme sparsity of current smokers in the very low/absent PPS-9 category in the present dataset.

Prospective intervention studies may eventually be useful, but they should follow rather than precede stronger longitudinal evidence. Randomized studies could examine whether interventions targeting self-regulation, behavioral planning, physical activity, diet, or combinations of these characteristics are accompanied by changes in both procrastination measures and metabolic outcomes. Such studies would be necessary before any claim could be made that modifying procrastination itself changes cardiometabolic health. The present cross-sectional findings alone do not support such an inference.

Finally, replication in population-based cohorts outside the occupational setting is needed. The present study was restricted to active Spanish workers aged 18–69 years, and future studies should include unemployed and retired populations, individuals with greater chronic disease burden, older adults, and populations from different countries and sociocultural contexts. External replication would help determine whether the graded associations observed here represent a broader population pattern or are partly specific to the characteristics of this occupational cohort.

## 5. Conclusions

In this large occupational population, higher operational PPS-9 categories were consistently associated with less favorable lifestyle profiles and a higher prevalence of metabolic syndrome. Physical activity and Mediterranean diet adherence showed substantial statistical overlap with the cross-sectional association between procrastination and MetS, as reflected by the marked attenuation observed after their incorporation into the multivariable models. Nevertheless, a graded association with MetS remained after adjustment for sociodemographic and lifestyle-related characteristics and across multiple sensitivity analyses. These findings support the relevance of considering procrastination alongside lifestyle-related characteristics when describing cardiometabolic health profiles in working populations. The exceptionally low prevalence of MetS and current smoking in the very low/absent PPS-9 reference category resulted in unusually large odds ratios. Complementary adjusted prevalence-ratio analyses yielded smaller estimates, although substantial associations remained. The magnitude of these associations should therefore be interpreted cautiously, particularly given the sparse reference-category data and limited age overlap between operational PPS-9 categories. Given the cross-sectional design, no temporal, mediational, or causal relationships can be inferred, and longitudinal studies are required to establish the temporal relationships among procrastination, lifestyle behaviors, and metabolic health.

From a clinical and occupational health perspective, these findings support an integrated approach to cardiometabolic prevention that considers physical activity, dietary habits, and potential difficulties with behavioral self-regulation alongside established metabolic risk factors. Workers reporting greater procrastination-related behaviors may benefit from a more comprehensive assessment of lifestyle characteristics and access to evidence-based preventive counseling when indicated. However, the PPS-9 categories used in this study are not clinically validated screening thresholds, and the cross-sectional findings do not justify using procrastination scores to diagnose metabolic risk or predict future MetS. Longitudinal studies are needed before procrastination-specific interventions can be recommended for cardiometabolic risk reduction.

## Figures and Tables

**Table 1:** Characteristics of the study population according to operational PPS-9 category.

Characteristic	Very Low/Absent (*n* = 18,829)	Moderate (*n* = 41,122)	High (*n* = 26,818)	Very High (*n* = 5415)
Age, years	33.02 ± 6.47	35.02 ± 7.08	47.78 ± 7.97	56.38 ± 5.68
Sex				
Male	8741 (46.4)	25,327 (61.6)	17,253 (64.3)	4123 (76.1)
Female	10,088 (53.6)	15,795 (38.4)	9565 (35.7)	1292 (23.9)
Educational level				
Primary	8426 (44.8)	23,950 (58.2)	17,107 (63.8)	3515 (64.9)
Secondary	8665 (46.0)	14,942 (36.3)	8458 (31.5)	1679 (31.0)
University	1738 (9.2)	2230 (5.4)	1253 (4.7)	221 (4.1)
Social class				
I	1740 (9.2)	2296 (5.6)	1247 (4.6)	222 (4.1)
II	6558 (34.8)	9484 (23.1)	4966 (18.5)	982 (18.1)
III	10,531 (55.9)	29,342 (71.4)	20,605 (76.8)	4211 (77.8)
Physically active	15,972 (84.8)	20,869 (50.7)	7080 (26.4)	522 (9.6)
High Mediterranean diet adherence	14,748 (78.3)	19,772 (48.1)	6547 (24.4)	516 (9.5)
Current smoker	3 (0.02)	18,040 (43.9)	12,261 (45.7)	2306 (42.6)
BMI, kg/m^2^	22.30 ± 1.83	26.06 ± 4.14	27.93 ± 4.51	31.01 ± 4.57
Waist circumference, cm	75.20 ± 7.57	82.16 ± 10.18	85.37 ± 11.38	91.53 ± 11.17
Systolic BP, mmHg	113.03 ± 12.52	118.45 ± 14.11	125.54 ± 16.01	134.62 ± 18.17
Diastolic BP, mmHg	67.98 ± 8.96	71.85 ± 10.32	76.90 ± 10.62	81.32 ± 10.83
Fasting glucose, mg/dL	82.60 ± 10.53	85.15 ± 11.59	89.44 ± 13.09	95.55 ± 15.33
Triglycerides, mg/dL	80.31 ± 42.52	103.08 ± 64.32	131.07 ± 96.05	154.51 ± 96.65
HDL-C, mg/dL	55.56 ± 6.94	53.01 ± 6.70	49.34 ± 7.16	46.83 ± 7.46
Metabolic syndrome (JIS)	78 (0.4)	2123 (5.2)	4482 (16.7)	2022 (37.3)

Data are presented as mean ± standard deviation for continuous variables and *n* (%) for categorical variables. PPS-9 categories are operational descriptive groupings and do not represent clinically validated or diagnostic cut-offs. Physical activity was classified according to the IPAQ-SF threshold of ≥600 MET-min/week. High Mediterranean diet adherence was defined as MEDAS ≥ 9. Current smoking was analyzed as current smoker versus non-smoker, with the latter category including never and former smokers. MetS was defined according to the harmonized Joint Interim Statement (JIS) criteria. Abbreviations: PPS-9, 9-item Pure Procrastination Scale; BMI, body mass index; BP, blood pressure; HDL-C, high-density lipoprotein cholesterol; JIS, Joint Interim Statement; MetS, metabolic syndrome; IPAQ-SF, International Physical Activity Questionnaire–Short Form; MEDAS, Mediterranean Diet Adherence Screener. Social class was categorized according to the three-category occupational classification of the Spanish Society of Epidemiology (CSO-SEE12): class I, managers and professionals; class II, intermediate occupations and self-employed workers; and class III, manual workers.

**Table 2:** Adjusted associations between operational PPS-9 categories and lifestyle-related characteristics.

Lifestyle-Related Characteristic	Very Low/Absent (*n* = 18,829)	Moderate (*n* = 41,122)	High (*n* = 26,818)	Very High (*n* = 5415)
Physical activity				
Physically active, *n* (%)	15,972 (84.8)	20,869 (50.7)	7080 (26.4)	522 (9.6)
Adjusted OR (95% CI)	1.00 (reference)	0.19 (0.18–0.20)	0.07 (0.07–0.08)	0.02 (0.02–0.03)
*p*-value	—	<0.001	<0.001	<0.001
Mediterranean diet adherence				
High adherence, *n* (%)	14,748 (78.3)	19,772 (48.1)	6547 (24.4)	516 (9.5)
Adjusted OR (95% CI)	1.00 (reference)	0.27 (0.26–0.28)	0.11 (0.10–0.11)	0.04 (0.04–0.04)
*p*-value	—	<0.001	<0.001	<0.001
Current smoking				
Current smoker, *n* (%)	3 (0.02)	18,040 (43.9)	12,261 (45.7)	2306 (42.6)
Adjusted OR (95% CI)	1.00 (reference)	7542.87 (2431.60–23,398.12)	32,511.83 (10,470.25–100,954.53)	70,590.07 (22,685.55–219,653.42)
*p*-value	—	<0.001	<0.001	<0.001

Data are presented as *n* (%) and adjusted odds ratios (aORs) with 95% confidence intervals (CIs). Separate binary logistic regression models were fitted for physical activity (active vs. inactive), Mediterranean diet adherence (high vs. low adherence), and smoking status (current smoker vs. non-smoker). All models were adjusted for age, sex, educational level, and social class. The very low/absent operational PPS-9 category was used as the reference group. Non-smokers included never and former smokers. PPS-9 categories represent operational descriptive groupings and should not be interpreted as clinically validated or diagnostic cut-offs. Abbreviations: PPS-9, 9-item Pure Procrastination Scale; OR, odds ratio; CI, confidence interval.

**Table 3:** Sequential multivariable association between operational PPS-9 categories and metabolic syndrome.

Model	Very Low/Absent	Moderate OR (95% CI)	High OR (95% CI)	Very High OR (95% CI)
Model 1	1.00 (reference)	12.74 (10.15–15.98)	62.07 (49.34–78.08)	215.87 (169.53–274.86)
Model 2	1.00 (reference)	12.63 (10.06–15.85)	61.28 (48.70–77.11)	212.56 (166.89–270.73)
Model 3A	1.00 (reference)	4.47 (3.55–5.62)	13.74 (10.90–17.33)	37.42 (29.32–47.77)
Model 3B	1.00 (reference)	6.00 (4.78–7.55)	19.38 (15.39–24.42)	55.34 (43.40–70.55)
Model 3C	1.00 (reference)	13.99 (11.14–17.58)	73.31 (58.02–92.62)	265.22 (206.98–339.84)
Model 4	1.00 (reference)	4.10 (3.26–5.17)	11.67 (9.22–14.78)	30.89 (24.06–39.65)

Values are odds ratios (ORs) and 95% confidence intervals (CIs) obtained from binary logistic regression models for prevalent metabolic syndrome according to the harmonized Joint Interim Statement (JIS) criteria. The very low/absent operational PPS-9 category was used as the reference group. Model 1 was adjusted for age and sex. Model 2 was additionally adjusted for educational level and social class. Model 3A included Model 2 covariates plus physical activity (active vs. inactive). Model 3B included Model 2 covariates plus Mediterranean diet adherence (high vs. low adherence). Model 3C included Model 2 covariates plus smoking status (current smoker vs. non-smoker). Model 4 included Model 2 covariates plus physical activity, Mediterranean diet adherence, and smoking status simultaneously. Non-smokers included both never smokers and former smokers. PPS-9 categories represent operational descriptive groupings and should not be interpreted as clinically validated or diagnostic cut-offs. Abbreviations: PPS-9, 9-item Pure Procrastination Scale; OR, odds ratio; CI, confidence interval; JIS, Joint Interim Statement. All comparisons *p* < 0.001.

**Table 4:** Statistical attenuation of the association between operational PPS-9 categories and metabolic syndrome after incorporation of lifestyle-related variables.

Model Added to Model 2	Moderate (%)	High (%)	Very High (%)
Physical activity (Model 3A)	41.0	36.3	32.4
Mediterranean diet adherence (Model 3B)	29.3	28.0	25.1
Current smoking (Model 3C)	−4.0	−4.4	−4.1
All lifestyle-related variables (Model 4)	44.4	40.3	36.0

Statistical attenuation was calculated relative to Model 2 using the regression coefficients on the log-odds scale as: 100 × (β_Model 2 − β_Adjusted model)/β_Model 2. Positive values indicate a reduction in the magnitude of the estimated cross-sectional association after incorporation of the corresponding lifestyle-related variable(s), whereas negative values indicate an increase in the coefficient. Model 3A incorporated physical activity; Model 3B incorporated Mediterranean diet adherence; Model 3C incorporated current smoking; and Model 4 incorporated physical activity, Mediterranean diet adherence, and current smoking simultaneously. These attenuation estimates are descriptive and should not be interpreted as evidence of mediation or causal pathways. Because of the non-collapsibility of odds ratios and differences in model scaling, these coefficient-based percentages should not be interpreted as directly comparable contributions of lifestyle variables. Marginally standardized absolute risk differences are reported separately in Section 3.5 and Supplementary Table S3.

**Table 5:** Adjusted Predicted Probabilities of Metabolic Syndrome According to Operational PPS-9 Category and Lifestyle-Related Characteristics.

PPS-9 Category	Physically Inactive	Physically Active	Low Mediterranean Diet Adherence	High Mediterranean Diet Adherence
Very low/absent	2.46 (1.88–3.03); 2857; 69	0.05 (0.02–0.09); 15,972; 9	1.63 (1.25–2.02); 4081; 68	0.06 (0.02–0.10); 14,748; 10
Moderate	9.63 (9.23–10.03); 20,253; 2064	0.25 (0.19–0.32); 20,869; 59	8.98 (8.60–9.36); 21,350; 2057	0.30 (0.23–0.37); 19,772; 66
High	24.22 (23.47–24.98); 19,738; 4410	1.21 (0.93–1.49); 7080; 72	23.69 (22.95–24.44); 20,271; 4400	1.53 (1.20–1.87); 6547; 82
Very high	45.81 (43.90–47.72); 4893; 1995	6.78 (4.30–9.25); 522; 27	46.20 (44.29–48.11); 4899; 1986	9.50 (6.54–12.46); 516; 36
*p* for interaction	<0.001		<0.001	

Values are marginally standardized predicted prevalences of MetS (%) with 95% confidence intervals, followed by subgroup sample size (*n*) and observed MetS events. Predicted probabilities were averaged over the observed covariate distribution of the full analytical sample (*n* = 92,184). Confidence intervals were calculated using the delta method. Subgroup counts and MetS events refer to the observed data, whereas predicted prevalences are model-adjusted estimates.

**Table 6:** Sensitivity Analyses of the Association Between Operational PPS-9 Categories and Metabolic Syndrome.

Sensitivity Analysis	*n*	Moderate OR (95% CI)	High OR (95% CI)	Very High OR (95% CI)
Primary analysis (JIS)	92,184	4.10 (3.26–5.17)	11.67 (9.22–14.78)	30.89 (24.06–39.65)
Model 4 with flexible age adjustment (cubic spline, 4 df)	92,184	4.17 (3.31–5.25)	14.33 (11.31–18.15)	50.33 (39.05–64.86)
Physical activity + diet, without smoking	92,184	4.27 (3.40–5.38)	12.85 (10.19–16.21)	34.77 (27.23–44.38)
MetS defined by ATP III	92,184	2.86 (2.23–3.67)	7.31 (5.67–9.43)	15.78 (12.05–20.66)
MetS defined by IDF	92,184	17.00 (10.51–27.48)	66.18 (40.80–107.34)	248.36 (151.88–406.14)
Age 40–49 years	27,153	4.16 (2.86–6.04)	14.84 (10.14–21.72)	65.19 (42.94–98.98)
Men	55,444	4.76 (3.44–6.58)	12.81 (9.22–17.79)	32.59 (23.15–45.87)
Women	36,740	3.64 (2.61–5.09)	12.28 (8.65–17.43)	37.66 (25.66–55.27)
Excluding diagnosed diabetes	91,491	4.23 (3.35–5.35)	11.83 (9.30–15.04)	29.89 (23.18–38.55)
Additional adjustment for BMI	92,184	1.94 (1.53–2.45)	3.65 (2.85–4.66)	5.96 (4.57–7.77)

Values are odds ratios (ORs) and 95% confidence intervals (CIs) for prevalent metabolic syndrome across operational PPS-9 categories, with the very low/absent category as the reference. Unless otherwise specified, models were adjusted for age, sex, educational level, social class, physical activity, Mediterranean diet adherence, and current smoking, corresponding to the covariate structure of Model 4. The additional physical activity and Mediterranean diet model excluded smoking status but retained age, sex, educational level, social class, physical activity, and Mediterranean diet adherence (*n* = 92,184). The flexible-age sensitivity model retained all Model 4 covariates but replaced the linear age term with a cubic spline with four degrees of freedom. Sex was omitted from the corresponding sex-stratified models. The age-restricted analysis included participants aged 40–49 years. The BMI sensitivity model additionally incorporated body mass index into Model 4. Alternative MetS definitions were based on ATP III and IDF criteria. PPS-9 categories represent operational descriptive groupings and should not be interpreted as clinically validated or diagnostic cut-offs. Abbreviations: PPS-9, 9-item Pure Procrastination Scale; MetS, metabolic syndrome; OR, odds ratio; CI, confidence interval; JIS, Joint Interim Statement; ATP III, Adult Treatment Panel III; IDF, International Diabetes Federation; BMI, body mass index.

**Table 7:** Number and prevalence of metabolic syndrome cases by PPS-9 category according to JIS, ATP III, and IDF definitions.

PPS-9 Category	Total *n*	JIS, *n* (%)	ATP III, *n* (%)	IDF, *n* (%)
Very low/absent	18,829	78 (0.41)	67 (0.36)	17 (0.09)
Moderate	41,122	2123 (5.16)	1540 (3.74)	1660 (4.04)
High	26,818	4482 (16.71)	3318 (12.37)	3543 (13.21)
Very high	5415	2022 (37.34)	1441 (26.61)	1783 (32.93)
Total	92,184	8705 (9.44)	6366 (6.91)	7003 (7.60)

IDF cases were recalculated using European waist circumference thresholds (≥94 cm in men and ≥80 cm in women).

## Data Availability

Privacy and Data Protection. Participant confidentiality and data security were maintained throughout the study. All identifying information was removed before analysis, and the analytical dataset was anonymized so that individual participants could not be identified. Data were processed in accordance with Spanish Organic Law 3/2018 on Personal Data Protection and Guarantee of Digital Rights and the European Union General Data Protection Regulation (Regulation [EU] 2016/679). Availability of Data and Materials. The dataset supporting the findings of this study is stored in a secure institutional repository at ADEMA University School. Anonymized data may be made available upon reasonable request to the corresponding author, subject to compliance with applicable ethical requirements, institutional policies, and European data protection regulations.

## Supplementary Materials

The following supporting information can be downloaded at: https://www.mdpi.com/article/10.3390/nu18213678/s1, Section S1.1. Description of the questionnaire; Section S1.2. Questionnaire administered to participants; Section S1.3. Scoring and operational classification; Section S1.4. Conceptual grouping of questionnaire ítems; Section S1.5. Conceptual correspondence with the original Pure Procrastination Scale; Table S1. Operational classification of procrastination scores; Table S2. Conceptual correspondence between the Spanish-language questionnaire used in this study and the original PPS [37]; Table S3. Marginally standardized predicted probabilities of metabolic syndrome and absolute risk differences by operational PPS-9 category across sequential logistic regression models; Table S4. Associations Between Operational PPS-9 Categories and Individual Components of Metabolic Syndrome; Figure S1. Participant flow diagram.
