# Supplementary material for: The Role of Lifestyle Behaviors in the Association Between Behavioral Procrastination and Metabolic Syndrome: A Cross-Sectional Study of 92,184 Workers
Source: Nutrients. 2026 Nov 6;18(21):3678. doi: 10.3390/nu18213678 (PMC13765348; doi:10.3390/nu18213678)

Supplementary Material S1

Spanish-Language Version of the Nine-Item Procrastination Questionnaire Administered to Study Participants

S1.1. Description of the questionnaire

Behavioral procrastination was assessed using a nine-item Spanish-language questionnaire adapted from the conceptual content of the Pure Procrastination Scale (PPS), prepared for use during routine occupational health examinations. The translation process involved a psychologist and another professional.

The questionnaire reproduced below contains the Spanish-language instructions, response options, and nine statements used to assess self-reported procrastination-related behaviors in the study population.

The Spanish-language version has not undergone formal psychometric validation in this occupational population. The score categories described below were used as operational classifications rather than clinically validated thresholds.

S1.2. Questionnaire administered to participants

Cuestionario de Procrastinación PPS-9 (Versión en español)

Instrucciones para el participante:

A continuación se presentan una serie de afirmaciones. Indica hasta qué punto estás de acuerdo o en desacuerdo con cada una de ellas, según lo que habitualmente haces, piensas o sientes.

Escala de respuesta (Likert de 5 puntos):

1 = Completamente en desacuerdo

2 = En desacuerdo

3 = Ni de acuerdo ni en desacuerdo

4 = De acuerdo

5 = Completamente de acuerdo

Ítems

1. A menudo pospongo decisiones importantes.
2. A menudo no empiezo una tarea hasta que no es absolutamente necesario.
3. A veces dejo tareas sin terminar por haberme demorado demasiado.
4. Pospongo decisiones hasta que es demasiado tarde.
5. Me cuesta comenzar a trabajar en cosas que me resultan difíciles o desagradables.

6. A menudo dejo pasar fechas límite o entrego cosas tarde.
7. Me doy cuenta de que debería estar haciendo algo, pero sigo postergándolo.
8. Me demoro innecesariamente en comenzar tareas, incluso si son importantes.
9. Frecuentemente me comprometo a hacer cosas que luego no cumplo a tiempo.

S1.3. Scoring and operational classification

The total score was calculated by summing the responses to the nine items, yielding a possible range from 9 to 45 points. Higher scores indicated greater self-reported procrastination-related behaviors.

Table S1. Operational classification of procrastination scores

**Total score**	**Operational category**
9–17	Very low/absent
18–26	Moderate
27–35	High
36–45	Very high

These categories were used for descriptive and statistical analyses and should not be interpreted as validated clinical or diagnostic cut-off points.

S1.4. Conceptual grouping of questionnaire items

The questionnaire documentation identified three conceptual groupings of procrastination-related behaviors:

**Conceptual domain**	**Item numbers**
Postponement of decisions	1, 4, 9
Delay in task initiation	2, 5, 8
Failure to meet deadlines	3, 6, 7

These groupings reflect the organization described in the questionnaire documentation and should not be interpreted as empirically validated subscales or an established factorial structure in the study population.

S1.5. Conceptual correspondence with the original Pure Procrastination Scale

The original Pure Procrastination Scale (PPS; Steel, 2010) comprises 12 items. The nine-item Spanish-language questionnaire administered in the present study was compared with the original PPS item content to describe the degree of conceptual correspondence.

Table S2. Conceptual correspondence between the Spanish-language questionnaire used in this study and the original PPS.

Study item	Spanish-language item administered	Closest original PPS item(s)	Assessment of correspondence
1	A menudo pospongo decisiones importantes.	PPS 1: Delaying decisions until it is too late	Partial: both concern decision postponement, but the original specifies a consequence
2	A menudo no empiezo una tarea hasta que no es absolutamente necesario.	PPS 5: Delaying tasks for days	Approximate: both concern delayed task execution, but the wording and timing differ
3	A veces dejo tareas sin terminar por haberme demorado demasiado.	PPS 10: Not completing things on time	Partial: both concern unsuccessful task completion, but the study item explicitly attributes it to delay
4	Pospongo decisiones hasta que es demasiado tarde.	PPS 1: Delaying decisions until it is too late	Close conceptual correspondence
5	Me cuesta comenzar a trabajar en cosas que me resultan difíciles o desagradables.	PPS 8: Delaying the start of work	Partial: the study item introduces task difficulty or unpleasantness, which is not specified in the original
6	A menudo dejo pasar fechas límite o entrego cosas tarde.	PPS 10–11: Not completing things on time / difficulty meeting deadlines	Close conceptual correspondence
7	Me doy cuenta de que debería estar haciendo algo, pero sigo postergándolo.	PPS 7: Repeatedly postponing tasks until tomorrow	Approximate: both concern continued postponement, but the wording differs
8	Me demoro innecesariamente en comenzar tareas, incluso si son importantes.	PPS 8: Delaying the start of work	Close conceptual correspondence
9	Frecuentemente me comprometo a hacer cosas que luego no cumplo a tiempo.	PPS 10–11: Not completing things on time / difficulty meeting deadlines	Partial: the study item additionally refers to commitments

Note: PPS item numbers refer to the original 12-item instrument. Correspondences were assessed retrospectively on the basis of item content and should not be interpreted as evidence of linguistic equivalence or psychometric validity. Several study items correspond conceptually to the same original PPS item, and some contain additional or modified content. Consequently, the questionnaire used in this study should be described as a nine-item Spanish-language adaptation informed by the PPS, rather than as a verified item-for-item translation of a nine-item original scale.

References

Steel, P. (2010). Arousal, avoidant, and decisional procrastinators: Do they exist? *Personality and Individual Differences, 48*, 926–934. https://doi.org/10.1016/j.paid.2010.02.025

Guilera, G., López-Martínez, M. D., Barrios, M., Hidalgo, M. D., Steel, P., & Gómez-Benito, J. (2024). Spanish validation of the pure procrastination scale: Dimensional structure, internal consistency, temporal stability, gender invariance, and relationships with personality and satisfaction with life. *Frontiers in Psychology, 14*, 1268855. https://doi.org/10.3389/fpsyg.2023.1268855

Measurement considerations

The analytical dataset retained the final operational procrastination category but did not retain individual item responses or continuous total scores. Therefore, retrospective calculations of internal consistency coefficients, exploratory or confirmatory factor analyses, and analyses using continuous PPS-9 scores were not possible.

The questionnaire and its operational classification procedure have previously been described in a publication involving the same occupational population (Sarmiento-Cruz et al., *Endocrines*, 2026; 7:60; doi:10.3390/endocrines7040060). This previous use does not constitute independent psychometric validation of the Spanish-language questionnaire.

Supplementary Table S3. Marginally standardized predicted probabilities of metabolic syndrome and absolute risk differences by operational PPS-9 category across sequential logistic regression models.

Analytical sample: 92,184 workers. All probabilities and risk differences are expressed as percentages or percentage points, respectively.

Model	PPS-9 category	Reference probability (%)	Category probability (%)	Risk difference (pp)	95% CI (pp)
Model 2	Moderate	0.39	4.69	4.30	4.08–4.52
Model 2	High	0.39	19.04	18.65	18.00–19.30
Model 2	Very high	0.39	44.24	43.85	41.98–45.71
Model 3A	Moderate	1.20	4.96	3.76	3.42–4.09
Model 3A	High	1.20	12.73	11.54	11.07–12.00
Model 3A	Very high	1.20	24.77	23.57	22.48–24.67
Model 3B	Moderate	0.89	4.94	4.05	3.77–4.34
Model 3B	High	0.89	13.36	12.47	12.02–12.91
Model 3B	Very high	0.89	26.91	26.02	24.85–27.19
Model 3C	Moderate	0.35	4.69	4.34	4.12–4.55
Model 3C	High	0.35	20.15	19.80	19.06–20.53
Model 3C	Very high	0.35	46.79	46.44	44.47–48.40
Model 4	Moderate	1.35	5.11	3.76	3.40–4.13
Model 4	High	1.35	12.30	10.95	10.45–11.45
Model 4	Very high	1.35	23.66	22.31	21.17–23.44

Model 2: age, sex, educational level, and social class. Model 3A: Model 2 plus physical activity (active/inactive). Model 3B: Model 2 plus Mediterranean diet adherence (high/low). Model 3C: Model 2 plus current smoking (current/non-current). Model 4: Model 2 plus all three lifestyle-related variables. The very low/absent PPS-9 category is the reference for each model.

Predicted probabilities were averaged over the same analytical sample after assigning each participant to the specified PPS-9 category while retaining their observed covariate values. Risk differences equal the standardized probability for the indicated PPS-9 category minus that for the very low/absent reference category. The 95% confidence intervals for risk differences were estimated using the delta method. Differences between nested models are descriptive, not causal or mediated effects. Smoking-adjusted results warrant caution because only three current smokers were observed in the very low/absent PPS-9 category.

To further characterize the association between operational PPS-9 categories and metabolic syndrome, exploratory analyses were conducted for each of the five individual components defined by the Joint Interim Statement (JIS) criteria. Adjusted prevalence ratios and 95% confidence intervals were estimated using modified Poisson regression with robust variance estimation, controlling for age, sex, educational level, social class, physical activity, Mediterranean diet adherence, and current smoking. These analyses aimed to identify differences in the patterns of association across metabolic components rather than to establish their causal contributions to the composite MetS outcome. The results are presented in Supplementary Table S4.

Supplementary Table S4. Associations Between Operational PPS-9 Categories and Individual Components of Metabolic Syndrome

JIS component	Moderate	High	Very high
Elevated waist circumference	4.63 (4.26–5.03)	11.87 (10.88–12.96)	27.22 (24.80–29.89)
Elevated triglycerides	0.67 (0.63–0.70)	0.69 (0.65–0.73)	0.76 (0.71–0.81)
Reduced HDL-C	0.61 (0.58–0.65)	0.69 (0.64–0.73)	0.72 (0.66–0.78)
Elevated blood pressure	1.53 (1.47–1.59)	2.04 (1.94–2.14)	2.46 (2.32–2.61)
Elevated fasting glucose	1.44 (1.34–1.55)	1.91 (1.75–2.09)	2.35 (2.11–2.62)

The very low/absent operational PPS-9 category served as the reference (aPR = 1.00). Adjusted prevalence ratios were estimated using modified Poisson regression with robust sandwich variance estimation, adjusting for age, sex, educational level, social class, physical activity, Mediterranean diet adherence, and current smoking (Model 4 covariates). Individual metabolic abnormalities were defined according to the Joint Interim Statement (JIS) criteria. These component-specific analyses were exploratory and should not be interpreted as causal effects or as estimates of the relative contribution of individual components to the composite MetS outcome.

Supplementary Figure S1. Participant flow diagram. Flow of participants from the initial occupational health database to the final analytical sample. A total of 2,143 records were excluded because of incomplete information on one or more variables required for the analyses. The final sample comprised 92,184 workers (97.7% of the initial database).

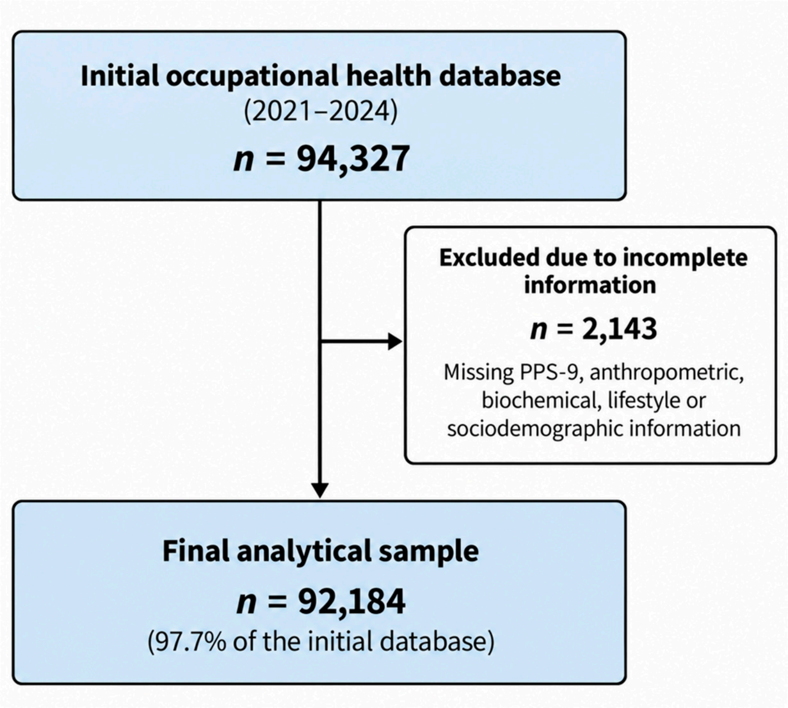

Supplement: Supplementary file 1 [file nutrients-18-03678-s001.zip › nutrients-4645999-supplementary.pdf]
